# Fungal Bioremediation of Selenium-Contaminated Industrial and Municipal Wastewaters

**DOI:** 10.3389/fmicb.2020.02105

**Published:** 2020-09-08

**Authors:** Mary C. Sabuda, Carla E. Rosenfeld, Todd D. DeJournett, Katie Schroeder, Karl Wuolo-Journey, Cara M. Santelli

**Affiliations:** ^1^Department of Earth and Environmental Sciences, University of Minnesota, Minneapolis, MN, United States; ^2^BioTechnology Institute, University of Minnesota, Saint Paul, MN, United States; ^3^Section of Minerals and Earth Sciences, Carnegie Museum of Natural History, Pittsburgh, PA, United States; ^4^Geosyntec Consultants, Minneapolis, MN, United States; ^5^Department of Ecology, Evolution, and Behavior, University of Minnesota, Saint Paul, MN, United States

**Keywords:** selenium, wastewaters, fungi, bioremediation, mycoremediation, ascomycetes, selenate, selenite

## Abstract

Selenium (Se) is an essential element for most organisms yet can cause severe negative biological consequences at elevated levels. The oxidized forms of Se, selenate [Se(VI)] and selenite [Se(IV)], are more mobile, toxic, and bioavailable than the reduced forms of Se such as volatile or solid phases. Thus, selenate and selenite pose a greater threat to ecosystems and human health. As current Se remediation technologies have varying efficiencies and costs, novel strategies to remove elevated Se levels from environments impacted by anthropogenic activities are desirable. Some common soil fungi quickly remove Se (IV and VI) from solution by aerobic reduction to solid or volatile forms. Here, we perform bench-scale culture experiments of two Se-reducing Ascomycota to determine their Se removal capacity in growth media conditions containing either Se(IV) or Se(VI) as well as in Se-containing municipal (∼25 μg/L Se) and industrial (∼2000 μg/L Se) wastewaters. Dissolved Se was measured throughout the experiments to assess Se concentration and removal rates. Additionally, solid-associated Se was quantified at the end of each experiment to determine the amount of Se removed to solid phases (e.g., Se(0) nanoparticles, biomass-adsorbed Se, or internal organic selenoproteins). Results show that under optimal conditions, fungi more efficiently remove Se(IV) from solution compared to Se(VI). Additionally, both fungi remove a higher percentage of Se from the filtered municipal wastewater compared to the industrial wastewater, though cultures in industrial wastewater retained a greater amount of solid-associated Se. Additional wastewater experiments were conducted with supplemental carbohydrate- or glycerin-based carbon products and additional nitrogen- and phosphorous-containing nutrients in some cases to enhance fungal growth. Relative to unamended wastewater experiments, supplemental carbohydrates promote Se removal from municipal wastewater but minimally impact industrial wastewater removal. This demonstrates that carbon availability and source impacts fungal Se reduction and removal from solution. Calculations to assess the leaching potential of solid-associated Se from fungal biomass show that wastewater Se release will not exceed regulatory limits. This study highlights the considerable potential for the mycoremediation of Se-contaminated wastewaters.

## Introduction

**FIGURE 1:**
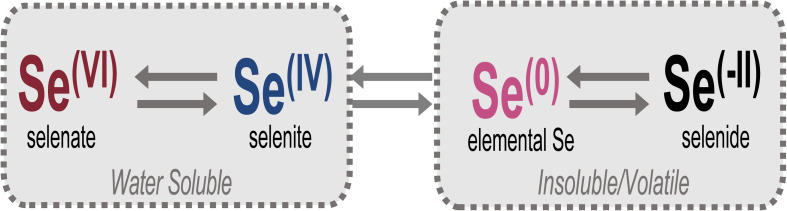
Relevant oxidation states of Se in the environment. Selenate [Se(VI)] and selenite [Se(IV)] oxyanions comprise total aqueous Se and are the forms targeted for bioremediation, largely through reduction to insoluble elemental Se [Se(0)] or volatile/insoluble selenide [Se(-II)] compounds.

Selenium (Se) is an essential trace element for most life yet is an element of increasing environmental concern due to elevated levels generated by anthropogenic activities and its toxicity to animals at these higher concentrations. In natural systems, Se can exist as volatile or non-volatile organic and solid-phase inorganic selenide [Se(-II)] compounds, nanoparticulate elemental Se(0), or aqueous selenite [Se(IV)] or selenate [Se(VI)] oxyanions (Figure 1). The speciation of Se controls its bioavailability, toxicity, and also its mobility through the environment (Ralston et al., 2008; Sharma et al., 2015). For example, the more oxidized forms (e.g., IV, VI) are generally more soluble and toxic (Lenz and Lens, 2009; Nancharaiah and Lens, 2015a; Chan et al., 2018). In humans and other animal life, chronic exposure to Se in elevated quantities can bioaccumulate and cause detrimental and severe biologic repercussions (Ohlendorf et al., 1986; Lemly, 1993, 1997, 2018; Goldhaber, 2003; Ty Lindberg et al., 2011; Nancharaiah and Lens, 2015b; Bailey, 2017).

Selenium is distributed naturally around the world through volcanic activity and subsequent atmospheric circulation (Kessi et al., 1999; Winkel et al., 2012; Tan L. C. et al., 2016), and high concentrations of Se are inherently found in some sedimentary rocks such as limestones and shales. Unearthing Se-containing shale beds and other geogenic rock sources through mining has been shown to release high concentrations of Se to the surface environment (Lemly, 2004; Zhu et al., 2008, 2012; Stillings and Amacher, 2010; Diehl et al., 2012; Khamkhash et al., 2017; Rosenfeld et al., 2018). Because Se is enriched in coal, anthropogenic activities involving coal, such as mining, processing, and combustion for electricity are especially problematic for increasing environmental Se concentrations to hazardous levels (Dreesen et al., 1977; Fernández-Martínez and Charlet, 2009; Dai et al., 2012; Zhu et al., 2012; Santos et al., 2015; Schwartz et al., 2018; Brandt et al., 2019).

Elevated selenium levels can also be found in areas related to the manufacturing of glass, ceramics, solar cells, and pharmaceuticals (Yamamura et al., 2007; Srivastava and Mukhopadhyay, 2013; Santos et al., 2015; Sharma et al., 2015; Tan L. C. et al., 2016; Bailey, 2017; Hadar et al., 2019). Se can further be an issue in the disposal and recycling of batteries and other electronic waste products (Garlapati, 2016). Reported Se concentrations of contaminated systems reach up to 1.4 mg/L in irrigation or agricultural wastewaters, 4.9 mg/L in oil refinery wastewater, 7 mg/L in lead wastewater, and 33 mg/L in gold mine wastewaters (Lemly, 2004; Astratinei et al., 2006; Santos et al., 2015; Tan L. C. et al., 2016). For comparison, the United States Environmental Protection Agency (USEPA) 2016 Se water quality guidelines for lentic and lotic aquatic systems are 1.5 and 3.1 μg/L (30 day chronic exposures), respectively (US EPA, 2016).

Several techniques exist for removing selenium from drinking water and industrial wastewater sources. Each approach is effective under certain conditions (e.g., pH, oxygen levels, Se form and concentration), and nearly all are costly. The most common chemical techniques include ferrihydrite adsorption (Rosengrant and Fargo, 1990) and tailored ion exchange (Boegel and Clifford, 1986; Twidwell et al., 1999). Biologically induced Se transformations (e.g., reduction, methylation, etc.) are ubiquitous throughout multiple domains of life, and Se bioremediation approaches can capitalize on this inherent activity. Previous studies have shown that diverse bacteria can transform Se(VI and IV) to elemental selenium (Oremland et al., 1989; Tomei et al., 1995; Zhang et al., 2008; Ikram and Faisal, 2010; Debieux et al., 2011; Tugarova et al., 2014; Staicu et al., 2015b; Medina Cruz et al., 2018). Biotic Se remediation strategies have focused largely on elemental selenium formation by bacteria (Lawson and Macy, 1995; Cantafio et al., 1996; Soda et al., 2011; Tan Y. et al., 2016) as the solid form of Se is insoluble, less bioavailable, and capable of physical separation from the treated wastewaters (Lenz et al., 2008; Soda et al., 2011; Winkel et al., 2012; Nancharaiah and Lens, 2015b; Tan L. C. et al., 2016; Wadhwani et al., 2016). Biomethylation of Se is also a very promising bioremediation technique, as the most commonly formed Se compound, dimethylselenide (DMSe), is non-toxic and volatile, which permanently leaves an open system (Thompson-Eagle and Frankenberger, 1990; Thompson-Eagle and Frankenberger, 1991; Frankenberger and Arshad, 2001). Some bioremediation strategies utilize Se(VI)-reducing bacteria, or a consortium of anaerobic Se(VI or IV)-reducing bacteria in bioreactors (Soda et al., 2011) to generate elemental Se. Complications often arise in the former strategy as Se(VI) reduction has been shown at times to result in the accumulation of Se(IV) (Fujita et al., 1997; Schröder et al., 1997). The latter strategy is also complicated due to inhibitory effects of other energetically favorable electron acceptors (e.g., nitrate or sulfate) typically abundant in wastewater (Steinberg et al., 1992; Lenz et al., 2008; Lai et al., 2014). If inorganic mercury is also present with Se in the wastewater, contemporaneous reduction of sulfate in these anaerobic bioreactors can lead to methylmercury production, a highly toxic compound that requires mitigation. Further, these approaches are efficient only if the anoxic conditions of the system are maintained. If oxygen is introduced, bacterial transformations of obligate anaerobic pathways can be inhibited (Oremland et al., 1989). Some bacteria can reduce Se aerobically (Dhanjal and Cameotra, 2010; Kuroda et al., 2011; Kagami et al., 2013; Staicu et al., 2015a), however, this application in bioremediation technology has been fairly limited to date and could benefit from research directed at the strengths and limitations of this approach.

While most knowledge of microbially mediated Se transformations is related to bacterial processes, previous studies have shown that common environmental fungi are capable of reducing Se oxyanions under atmospheric conditions (Gharieb et al., 1995; Rosenfeld et al., 2017, 2018, 2020; Kieliszek, 2018; Liang et al., 2019). Fungal cells have been shown to produce many more intracellular nanoparticles of Se than the average bacterium capable of Se reduction (e.g., Debieux et al., 2011; Butler et al., 2012; Rosenfeld et al., 2017) and are associated with higher quantities of extracellularly bound Se(0). Even in oxic environments, biogenic elemental Se(0) is stable and extremely slow to re-oxidize (Losi and Frankenberger, 1997; Dowdle and Oremland, 1998; Minaev et al., 2005). The Se(0) biominerals are encapsulated in proteins, which are hypothesized to aid in nanoparticle stability (Xu et al., 2018) and to control their size and morphology (Dobias et al., 2011). To this end, mycoremediation strategies may be a viable route for removing high concentrations of Se from contaminated environments, yet to date minimal work has been done to examine this.

To directly assess the aerobic mycoremediation potential of Se-contaminated wastewaters, laboratory experiments were conducted with two Se-reducing fungal isolates grown in Se-containing municipal (WWM) and industrial (WWI) wastewaters to measure total Se loss with respect to time, Se form (selenite or selenate), fungal species, and fungal growth stimulation with various carbon sources. The fungal isolates include *Alternaria alternata* strain SRC1lrK2f (Rosenfeld et al., 2017), originally isolated from a coal mine drainage treatment system (Santelli et al., 2010), and *Alternaria* sp. strain CMED5rs1aP4, an organism cultured from Se-enriched soil overlying the Phosphoria Formation at a reclaimed mining site in Idaho, United States (Rosenfeld et al., 2018). These experiments were conducted alongside positive controls — both fungal species grown in synthetic Se-containing growth media — for comparison. The aim of these experiments was to identify the selenium removal capacity of these fungal species when grown under ideal conditions and exposed to Se concentrations that emulated the wastewaters obtained for this study. In these experiments, the media contained nutrients that promote fungal growth, reduction, and removal of dissolved Se(IV) or Se(VI) compounds (Rosenfeld et al., 2017), but are free of any growth or reduction inhibitors (biological or chemical) that might be present in natural wastewaters. Additional short-term (7 day) experiments were conducted with proprietary carbon sources, MicroC^®^ 4000 (carbohydrate-based) and MicroC^®^ 2000 (glycerin-based), and nitrogen (N) and phosphorus (P) supplements to assess whether these specific carbon amendments aided in the removal of Se from contaminated waters. Based on these data, we gain insight and a better understanding of the potential for efficient and cost-effective fungal water treatment strategies for Se-contaminated environments.

## Materials and Methods

### Fungal Isolation

Two fungal strains were used in the current study. The first, *Alternaria alternata* strain SRC1lrK2f, was isolated and identified from a coal mine drainage impacted wetland in central Pennsylvania as previously described (Santelli et al., 2010). Original isolation of this strain was based on its capacity to oxidize manganese, however, the strain has also been confirmed to independently aerobically reduce Se oxyanions (Rosenfeld et al., 2017). The second strain was isolated from Se contaminated surface soils in southeast Idaho (Rosenfeld et al., 2018). To isolate this second strain, soil samples were collected in June 2015 using sterile field tools. Samples were stored throughout the duration of the field campaign and shipped at 4°C (∼ 5 days total). Culture enrichments were initiated within 5 days of collecting the samples. First, field moist soil was combined with sterile artificial freshwater (2 g/L NaCl, 0.4 g/L MgSO_4_⋅7H_2_O, 0.2 g/L CaCl_2_⋅2H_2_O, 0.13 g/L K_2_HPO_4_⋅3H_2_O) in a ratio of 1g soil:2 mL water and vortexed to mix. Serial dilutions to 1/10^–4^ were plated on three types of agar-solidified media amended with 20 mM HEPES (pH 7) and 100 μM Na_2_SeO_3_ or Na_2_SeO_4_: AY (Miyata et al., 2004), K (Templeton et al., 2005), or 1/2-strength potato dextrose agar (PDA). Se-tolerant and Se-transforming microorganisms were transferred to fresh media until they were deemed axenic. The isolate used in the current study (*Alternaria* sp. CMED5rs1aP4) was collected in the Champ Mine East Dump location 5, and the isolate originated from an enrichment culture using 1/10 dilution on PDA and 100 μM Na_2_SeO_3_.

Genomic DNA was extracted from the isolate and the ITS1-5.8S rRNA-ITS2 region (referred to as ITS) was amplified using primer pair ITS1F/ITS4 (Gardes and Bruns, 1993; Santelli et al., 2014). The resulting sequence was imported into the BLAST nucleotide search program (accessed February 2020; http://blast.ncbi.nlm.nih.gov/Blast.cgi), and sequences from 23 of the most closely related organisms were collected (99 to 100% sequence similarity). ARB (Ludwig et al., 2004) was used to align sequences (isolate and related organisms) and construct phylogenetic trees. Maximum Likelihood phylogenetic trees of the ITS regions of Se(IV/VI)-reducing fungi from this study and previous studies were constructed using the Randomized Axelerated Maximum Likelihood (RAxML) package in ARB with a General Time Reversible (GRTCAT) model and a rapid bootstrap analysis (1000 bootstraps).

### Wastewater Experiment Setup and Sampling

Bench scale pure-culture experiments were conducted with Se reducing fungal isolates *Alternaria alternata* strain SRC1lrK2f and *Alternaria* sp. strain CMED5rs1aP4. To achieve a consistent amount of biomass before starting each experiment, the fungi were first grown in individual 500 mL Erlenmeyer flasks under atmospheric conditions in triplicate with 250 mL of AY growth media (adapted from Miyata et al., 2004 as described in Rosenfeld et al., 2017) without selenium for 14 days. After this time, the growth media was removed via filtration using sterile 0.2 μm polyethersulfone (PES) bottle-top disposable filters, leaving the fungal biomass on the filter. Fresh Se-containing media or wastewater (50 mL) was then added into the top of the filter unit to resuspend the biomass. The new media and fungal biomass were then placed back into the original flask, and additional media or wastewater was added until the final volume reached 250 mL. This exchange of plain media with Se-containing growth media or wastewater signified the start of the experiments. All experiments were conducted in triplicate.

Wastewater treatments in the current study include filtered (0.2 μm) and unfiltered industrial wastewater (WWI; ∼2000 μg/L Se) and filtered and unfiltered municipal wastewater (WWM; ∼25 μg/L Se). Positive controls were prepared using AY growth media amended with approximately 2000 μg/L or 25 μg/L Se(IV) or Se(VI) provided as sodium selenite (Na_2_SeO_3_; Alfa Aesar) or sodium selenate (Na_2_SeO_4_; Acros Organics). As the growth media is autoclaved for sterility, an unknown volume of water loss commonly occurs, which can result in concentrations of Se and other sterile media components added post-autoclave to be slightly more concentrated than expected. The variations in starting Se concentration in the different media may not be uniform for all experiments, as experiments were performed at different times. Due to autoclave size restrictions, media were made in 5L bottle batches, and small differences in autoclave cycles may have influenced the water levels to different degrees when autoclaving. Triplicate 50 mL samples were collected from each batch of media or wastewater to determine starting Se concentration. Abiotic controls were also prepared using the two filtered wastewater types. AY growth media abiotic controls were not established for this study, as previous work has consistently identified negligible (i.e., ≤1%) Se removal in abiotic systems without fungi (Rosenfeld et al., 2017, 2020). Ethanol-killed biomass controls were also assembled for the two filtered wastewater types. For these, biomass was grown for two weeks in AY media as described above and collected on disposable 0.2 μm PES filters. The biomass was then rinsed with sterile ultrapure (18.2 MΩ) water, filtered again, and resuspended in sterile Falcon tubes containing 100% ethanol for 2 days. The empty flasks were autoclaved for 60 min to ensure sterility for these experiments. After 2 days, ethanol was removed, and biomass was rinsed with sterile selenium-free growth media to remove residual ethanol. The washing solution was then removed, and the biomass was placed back into its respective sterile flask with 250 mL of WWI or WWM as described above. Flasks were monitored to ensure no new fungal growth occurred after ethanol treatment.

Once the experiments were started (experiment day 0; biomass growth day 14), flasks were sampled using a draw-and-fill method at 0, 2, 7, and 21 days. On each sampling day, 50 mL from each flask was collected and filtered through a 0.2 μm disposable PES filter into a polyethylene sample bottle pre-filled with 1 mL concentrated trace-grade nitric acid (HNO_3_). After each sample was taken, 50 mL of the respective fresh media or wastewater was replaced to maintain consistent volume throughout the experiment.

### Wastewater Chemistry

Due to the confidential nature of WWI and WWM used in this study, only limited water chemistry data is available from onsite measurements. At the time measurements were taken, WWM had a total Se concentration of 25 μg/L and low levels of metal(loid)s such as copper (3.1 μg/L), nickel (2.4 μg/L), zinc (47.5 μg/L), arsenic (1.4 μg/L), lead (0.43 μg/L), and cadmium (0.2 μg/L). WWI, on the other hand, had a total Se concentration of 1987 μg/L (approximately 2000 μg/L), and was high in sulfate (35,033 mg/L), total dissolved solids (50,833 mg/L), and metal(loid)s including copper (447 μg/L), nickel (2357 μg/L), arsenic (60 μg/L), lead (40 μg/L), and cadmium (57.7 μg/L), among many others (Supplementary Table S1). The pH of both waters is approximately 6.5. It is important to note that the wastewaters were filtered prior to onset of the study (except for the unfiltered wastewater experiments), but the potential for Se to interact with abiotic or biotic components prior to filtration combined with the heterogeneous nature of the wastewater may have caused differences in the starting total Se concentration of the treatments.

### Carbon-Amended Experiments

To assess whether additional carbon amendments could enhance biomass growth and/or Se removal, separate 7-day culture experiments with MicroC^®^ 4000 (Environmental Operating Solutions, Inc., United States Patent #7,144,509/Canadian Patent #2,476,025; Boyd and Azad, 2006) or MicroC^®^ 2000 were conducted (Environmental Operating Solutions, Inc., United States; Ledwell and Togna, 2019). MicroC^®^ 4000 is an industry-approved carbohydrate-based carbon source commonly used in water treatment applications and optimized for drinking water applications, similar to the WWM chemistry described here. MicroC^®^ 2000 is a glycerin-based carbon source optimized for wastewater denitrification applications, similar to the WWI chemistry described here. As the carbon amendment products are proprietary, the exact composition is unknown, but the carbohydrate-based MicroC^®^ 4000 amendment is a clear, low viscosity fluid and the glycerin-based MicroC^®^ 2000 contains ∼71% glycerin, less than 0.1% methanol, and 0.3% fatty acid. In previous studies with these fungi, most Se removal occurred within approximately one week, depending on culture conditions (Rosenfeld et al., 2017, 2020). Additionally, 7 days is a more common water treatment timeline compared to 21 days. Cultures were established in duplicate and sampled as previously described but filled with 150 mL of fluid due to the finite quantity of wastewater remaining.

For the MicroC^®^ 4000 (carbohydrate) treatment, 3 mL of MicroC^®^ 4000 was added to 147 mL of wastewater in each flask to match the chemical oxygen demand (COD) of AY growth media (calculations provided in Supplementary Information text). For the MicroC^®^ 2000 (glycerin) treatment, 0.31 mL of MicroC^®^ 2000 was added to 150 mL of wastewater. Additionally, due to the satisfactory amount of nutrients in WWM, 1.5 mM K_2_HPO_4_ and 10 mM NH_4_Cl (final concentration) were supplemented to only WWI as P and N sources, respectively. Due to the minimal abiotic interactions of Se observed through previous studies and in the abiotic controls established for wastewater and glycerin-amended cultures in this study (described below), abiotic controls were not established for the carbohydrate-amended cultures.

Positive controls were also established using MicroC^®^ amendments. Media for these treatments was composed of 1 mM MgSO_4_, 10 mM NH_4_Cl, 1.5 mM K_2_HPO_4_/KH_2_PO_4_, 20 mL HEPES buffer (1 M, pH = 7, 4°C), and 1 mL trace element solution (Rosenfeld et al., 2017) and was amended with either 20.7 mL MicroC^®^ 4000 or 0.31 mL MicroC^®^ 2000 per liter media. Each of the MicroC^®^ amended growth media were also supplemented with either 25 μg/L Se(IV or VI) or 2000 μg/L Se(IV or VI). Abiotic (no biomass) controls were established for MicroC^®^ 2000 cultures and described below. These MicroC^®^-amended experiments are hereafter referred to generally as carbon-amended.

### Analytical Methods

#### Total Dissolved Se

Filtered samples (0.2 μm) from each experiment at each sampling point were collected into HNO_3_-spiked bottles prepared by Pace Analytical. Samples were stored at 4°C prior to total Se analysis via EPA method 6020 using Inductively Coupled Plasma Mass Spectroscopy (ICP-MS) at Pace Analytical in Minneapolis, MN.

#### Fungal Growth and Total Solid-Associated Se

To assess the amount of fungal growth and the amount of selenium associated with the solid phase, fungal biomass and any associated solid materials [e.g., bound extracellular Se(0) nanoparticles] in the flasks were filtered and collected at 28 days (7 days for carbon-amended experiments), after all draw-and-fill sampling was complete. Biomass was collected on pre-weighed 0.2 μm methyl cellulose ester (MCE) filters, air-dried overnight, and weighed. Subtraction of the preliminary (clean) filter weight from the combined filter and biomass weights provided the weight of fungal biomass reported here. To estimate the amount of biomass present at the start of the experiments (i.e., the point where Se-free AY growth media in cultures was replaced with respective Se-containing growth media or wastewater) and fungal growth over time, 4 flasks of each strain were grown in Se-free 250 mL AY growth media for 14 days. Biomass was then filtered onto a pre-weighed MCE filter, dried overnight, and weighed. The starting dry biomass weight (i.e., prior to Se addition) for *A. alternata* was 15 mg ± 2 and *Alternaria* sp. was 27 mg ± 1. Any weights greater than the dry biomass weights at experiment onset indicate additional fungal growth by experiment end. Samples were stored at 4°C until further processing.

Air-dried biomass and filters were digested along with the MCE filter in an individual perfluoroalkoxy (PFA) bottle (Thermo Fisher Scientific) by adding 4.5 mL trace metal grade nitric acid and 0.5 mL hydrogen peroxide, as described previously (Rosenfeld et al., 2017, 2020). Samples were diluted 1:1 and analyzed by Inductively Coupled Plasma Optical Emission Spectroscopy (ICP-OES) in the Analytical Geochemistry Laboratory at the University of Minnesota.

Unlike previous studies with these fungal isolates (e.g., Rosenfeld et al., 2017, 2020), mass balance calculations between aqueous- and solid-phase Se to obtain “volatile or missing Se” were not performed. Previous studies were conducted by sacrificing a set of flasks at each sampling point for aqueous- and solid- phase Se, respectively. The draw-and-fill method used here added Se at each sampling point, which could allow more Se to accumulate in biomass over time. Here, we use the same flasks throughout the entire experiment, so any mass balance calculations would not be comparable to aqueous Se removal.

### Statistical Analysis

For solid-associated Se and biomass weights in wastewater, we compared wastewater types (WWI, WWM), the two fungal species (*A. alternata*, *Alternaria* sp.) and five experiment types (unamended, unfiltered, carbohydrate-amended, glycerin-amended, killed controls) by two-way and three-way analysis of variance (ANOVA). We also compared solid-associated Se and biomass weights in the three media types (AY media, carbohydrate-amended, glycerin-amended), two fungal species, and Se starting condition [Se(IV or VI) at 25 or 2000 μg/L Se] by two-way, and three-way analysis of variance (ANOVA).

Tukey’s Honest Significant Difference (HSD) *post hoc* test was used to examine solid-associated Se and biomass weights data for significance of interactions within wastewater/media type, fungal species, and either experiment type or selenium starting condition. Statistical differences between wastewater/media types, fungal species, experiment type, and selenium starting condition were determined using a three-way ANOVA followed by Tukey’s HSD *post hoc* test to determine significance of each. Lowercase italic letters were placed on top of bar plots in respective figures to note statistical relationships. In a figure, bars with the same letter above (a, a) are not statistically different from each other, whereas bars with different letters (a, b) are statistically different. All statistical analyses were performed using the R basic statistics package R Core Team (2019). Results were considered significant at *p*-values < 0.05.

## Results

### Selenium Remediation Potential by Fungi in Growth Media

#### Fungal Isolates

Phylogenetic analysis of the Se-transforming isolate from Se-contaminated Idaho soils revealed the fungus is a member of the *Alternaria* genus but is phylogenetically distinct from the other isolate in this study, *Alternaria alternata* strain SRC1lrK2f (Supplementary Figure S1). Isolate CMED5rs1aP4 is most closely related (99–100% sequence identity in the ITS1-5.8S rRNA-ITS2 region) to three species of the *Alternaria* genus: *A. terricola, A. multiformis*, and *A. heterospora*. Because it is not phylogenetically distinct from these related species, the new isolate will be referred to hereafter as *Alternaria* sp. CMED5rs1aP4 (or *Alternaria* sp. in this study). The two isolates exhibited different morphologies – *Alternaria alternata* appeared to grow as one filamentous biomass, whereas *Alternaria* sp. grew as many small round (∼3–5 cm) biomass units with a dark pigmented center (Supplementary Figure S2).

#### AY Growth Media Experiments

**FIGURE 2:**
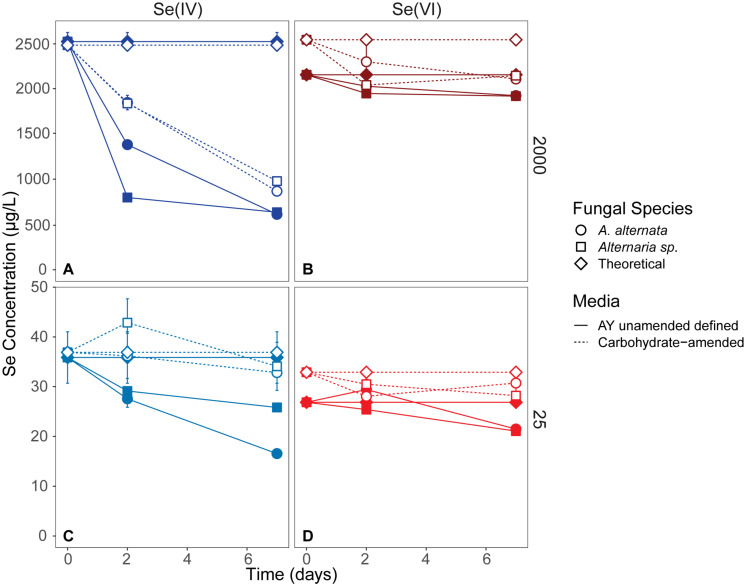
Aqueous Se concentrations (μg/L) remaining in solution over 7 days in AY growth media experiments (closed symbols, solid lines) and carbohydrate-amended experiments (open symbols, dashed lines) supplemented with ∼2000 μg/L Se(IV or VI) **(A,B)** and ∼25 μg/L Se (IV or VI) **(C,D)** concentrations. Both fungi remove Se(IV) and Se(VI) from solution, but are able to more quickly remove higher quantities of Se(IV) than when exposed to Se(VI). Error bars represent the standard deviation calculated from triplicates (AY growth media experiments) and duplicates (carbon-amended experiments). For duplicates, data points represent calculated means and error bars represent actual values measured for each duplicate. Symbols without error bars have a standard deviation that is smaller than the symbol size. Theoretical calculations were made using the starting concentration of total Se measured, and assuming that under ideal abiotic conditions, the amount and volume of Se removed during sampling would be replaced when adding fresh media or wastewater back into the flask. For day 21 data, see Table 1 and Supplementary Table S2.

Fungal growth experiments assessed the selenium removal capacity of our two fungal species when exposed to either low or high Se concentrations provided as Se(IV or VI). Unexpectedly, both fungi removed more Se when supplemented with higher concentrations, regardless of the Se oxidation state present (Table 1; Figure 2; Supplementary Table S2). After 21 days, *A. alternata* and *Alternaria* sp. grown in Se-supplemented growth media containing approximately 2000 μg/L Se(VI) (2153 μg/L actual measured concentration) both removed ∼200 μg/L Se (∼18%; Supplementary Table S2). Roughly half of all Se(VI) removed from solution was removed by the first 7 days in these higher concentration experiments, which mimic the total Se concentrations observed in WWI. Both fungal isolates, however, were considerably more effective in the removal of Se(IV) compared to Se(VI). After 7 days of exposure to high Se(IV) concentrations, *A. alternata* and *Alternaria* sp. removed ∼75% of the Se from solution down to ∼623 μg/L (Table 1; Supplementary Table S2).

**TABLE 1:** Concentrations and rates of Se removal in positive control growth media experiments with and without carbon amendments.

**Se Form and Concentration**	**Experiment Type**	**Fungal Species**	**Days**	**Se removed (%)**	**Rate (μg/L Se removed/day)**	**Amount removed by 21 days (μg/L)**	**Days to achieve standing water EPA limits (1.5 μg/L)**	**Days to achieve flowing water EPA limits (3.1 μg/L)**	**Solid Se/biomass (μg/mg)**
**25 μg/L Se(VI) (Municipal)**	AY growth media	*A. alternata*	7	22% ± 5	0.8 ± 0.2	18	29.8	27.9	-
		*Alternaria* sp.	7	22% ± 5	0.9 ± 0.2	18	29.8	27.9	-
		*A. alternata*	21	24% ± 2	0.3 ± 0.0	7	76.5	71.7	0.11
		*Alternaria* sp.	21	28% ± 7	0.4 ± 0.1	7	76.5	71.7	0.08
	Carbohydrate-amended media	*A. alternata*	7	6% ± 8	0.3 ± 0.3	6	110	105	0.00
		*Alternaria* sp.	7	15% ± 1	0.7 ± 0.0	15	44.1	41.9	0.00
	Glycerin-amended media	*A. alternata*	7	0% ± 3	0.0 ± 0.1	0	-	-	0.00
		*Alternaria* sp.	7	0% ± 2	0.0 ± 0.1	0	-	-	0.01
	Abiotic Controls	Glycerin-amended	7	0% ± 6	0.0 ± 0.2	0	-	-	-0.02
**25 μg/L Se(IV) (Municipal)**	AY growth media	*A. alternata*	7	53% ± 6	2.7 ± 0.2	57	12.7	12.1	-
		*Alternaria* sp.	7	28% ± 5	1.4 ± 0.2	30	24.2	23.0	-
		*A. alternata*	21	58% ± 1	1.0 ± 0.0	21	34.5	32.9	0.17
		*Alternaria* sp.	21	47% ± 6	0.8 ± 0.1	17	42.6	40.6	0.09
	Carbohydrate-amended media	*A. alternata*	7	11% ± 1	0.6 ± 0.1	12	62.1	59.3	0.01
		*Alternaria* sp.	7	8% ± 20	0.4 ± 1.0	9	82.8	79.1	0.03
	Glycerin-amended media	*A. alternata*	7	52% ± 5	2.1 ± 0.1	45	12.8	12.1	0.23
		*Alternaria* sp.	7	24% ± 2	1.0 ± 0.1	21	27.5	25.9	0.04
	Abiotic Controls	Glycerin-amended	7	7% ± 0	0.4 ± 0.0	6	-	-	0.15
**2000 μg/L Se(VI) (Industrial)**	AY growth media	*A. alternata*	7	11% ± 1	33.0 ± 3.7	693	65.2	65.1	-
		*Alternaria* sp.	7	11% ± 1	33.7 ± 2.4	708	63.8	63.8	-
		*A. alternata*	21	18% ± 1	18.0 ± 0.7	377	120	120	1.13
		*Alternaria* sp.	21	19% ± 1	19.4 ± 0.6	407	111	111	1.29
	Carbohydrate-amended media	*A. alternata*	7	17% ± 0	62.0 ± 0.0	1302	40.9	40.9	0.12
		*Alternaria* sp.	7	16% ± 2	56.7 ± 6.1	1191	44.8	44.7	0.15
	Glycerin-amended media	*A. alternata*	7	2% ±4	5.30 ± 12	111	378	378	0.16
		*Alternaria* sp.	7	5% ± 1	12.9 ± 2.0	270	155	155	0.19
	Abiotic Controls	Glycerin-amended	7	7% ± 1	21.0 ± 1.4	441	95.2	95.1	0.22
**2000 μg/L Se(IV) (Industrial)**	AY growth media	*A. alternata*	7	76% ± 3	273 ± 2.3	5724	9.2	9.20	-
		*Alternaria* sp.	7	75% ± 5	269 ± 4.1	5655	9.4	9.30	-
		*A. alternata*	21	78% ± 4	93.2 ± 1.1	1957	27.0	27.0	16.1
		*Alternaria* sp.	21	76% ± 1	90.6 ± 0.4	1903	27.8	27.8	12.3
	Carbohydrate-amended media	*A. alternata*	7	65% ± 1	230 ± 1.1	4839	10.8	10.7	5.02
		*Alternaria* sp.	7	61% ± 4	214 ± 4.9	4503	11.6	11.6	4.09
	Glycerin-amended media	*A. alternata*	7	31% ± 2	83.7 ± 4.7	1758	22.8	22.8	13.5
		*Alternaria* sp.	7	43% ± 3	118 ± 4.8	2472	16.2	16.2	7.61
	Abiotic Controls	Glycerin-amended	7	-1% ± 0	-	-	-	-	0.32
									

In cultures amended with a lower Se(VI) concentration (∼27 μg/L; similar to WWM) 21 μg/L Se remained in solution for both *A. alternata* and *Alternaria* sp. after 7 days, resulting in only a 22% removal (Figure 2). When placed in growth media with the lower Se(IV) starting concentration (34 μg/L), the two fungi removed 53% and 28% of the added Se, down to 17 and 26 μg/L, respectively, after 7 days. Due to the draw-and-fill method used in these experiments, the Se concentration would be expected to remain constant over the entire experimental time if no fungal-mediated or abiotic Se removal were occurring (referred to below as the “theoretical” Se concentration; see Figure 2).

#### Carbon-Amended Media Experiments

To assess whether carbon additions promoted fungal growth and Se removal from WWI and WWM, two industry-approved glycerin- and carbohydrate-based carbon supplements were tested. Since most Se removal occurred within the first 7 days of the AY growth media experiments, Se removal was only monitored for 7 days under these carbon-amended conditions. In these experiments the carbon-based products were added to each wastewater so that the final wastewater chemical oxygen demand (COD) was comparable to the COD of the AY growth media (as described in Supplemental Information text).

When amended with additional carbohydrates, Se(IV) removal from solution was much greater than Se(VI) for both *A. alternata* and *Alternaria* sp., similar to the results in AY growth media. In the experiments with the high (∼2500 μg/L) Se(IV) concentration and carbohydrate-based carbon source, *A. alternata* removed 65% and *Alternaria* sp. removed 61% of the Se over 7 days. When the low concentration of Se(IV) was added, *A. alternata* removed 11% and *Alternaria* sp. removed 8% Se(IV) (Table 1; Figure 2). This selenite removal in the presence of additional carbohydrates was slightly lower than the quantity of Se removed by the fungi in AY media described above (Table 1). In the carbohydrate-amended experiments containing Se(VI), *A. alternata* cultures exposed to high Se(VI) levels removed only 17% Se over 7 days, and isolate *Alternaria* sp. removed a similar amount (16%). In the low concentration Se(VI) experiments, *A. alternata* removed only 6% of Se over 7 days. In comparison, *Alternaria* sp. was more effective in Se(VI) removal where it removed 15% of Se (Table 1; Figure 2).

In contrast, the glycerin amendment did not allow for Se removal in all cases. When the fungal isolates were grown with high Se(VI) concentrations and glycerin, *A. alternata* cultures removed only 2% and *Alternaria* sp. removed 5% Se after 7 days. Both species performed poorly when grown with low Se(VI) concentrations where nearly all Se remained in solution at the end of the experiment. When selenite was substituted for selenate under these same high Se conditions, *A. alternata* removed 31% of the Se, and *Alternaria* sp. outperformed *A. alternata* by removing 43% Se from solution (Table 1; Supplementary Figure S3). In contrast, *A. alternata* outperformed *Alternaria* sp. in the low Se(IV) concentration experiments amended with glycerin. *A. alternata* removed 52% selenite compared to *Alternaria* sp., which only removed 24%. The abiotic controls established for low concentration Se(VI) and high concentration Se(IV) experiments did not show any removal of Se. In the high concentration Se(VI) and low concentration Se(IV) experiments, only a small percentage (7%) was removed over the 7 day timeline.

### Dissolved Selenium Remediation Potential by Fungi in Wastewater

#### Unamended Wastewater

**FIGURE 4:**
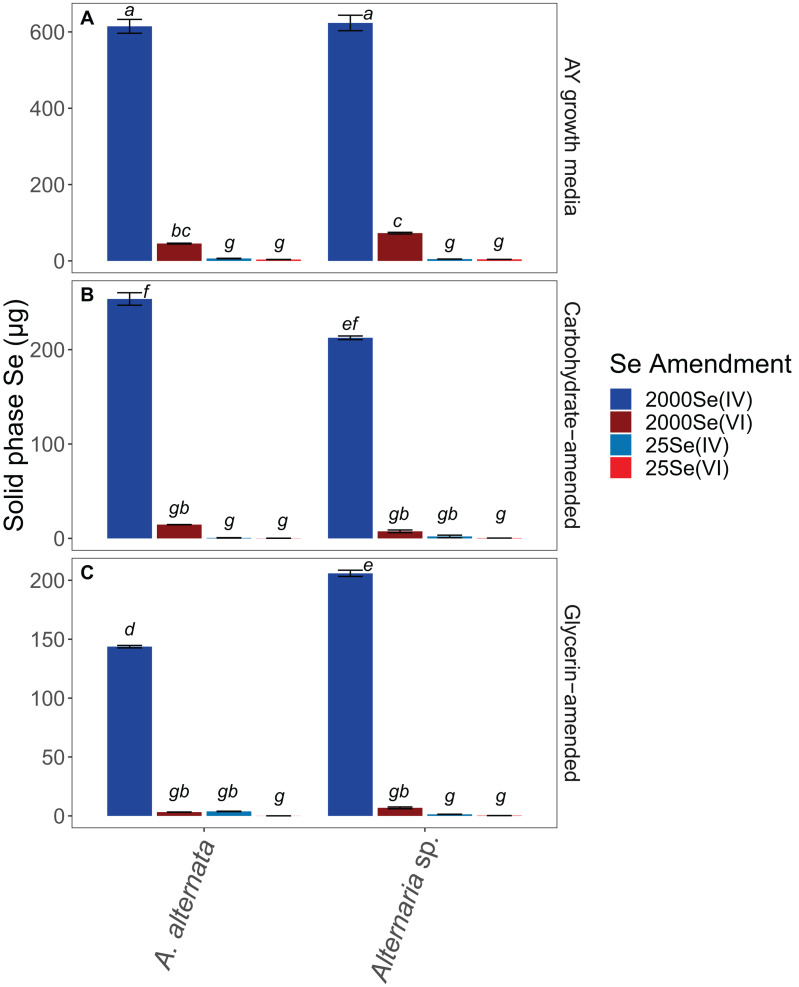
Solid associated Se concentrations for all growth media experiments. Note that AY growth media **(A)** ran for 21 days whereas carbon-amended experiments **(B,C)** ran for only 7 days before biomass was collected. Error bars represent the standard deviation calculated from triplicates (unamended wastewater experiments) and duplicates (carbon-amended experiments). Statistically different solid-associated Se accumulations (*p* < 0.05) among different treatments are indicated by different lowercase letters.

Cultures of *A. alternata* and *Alternaria* sp. were grown in both filtered (0.2 μm) and unfiltered wastewater. The filtered WWI measured at the start of the experiment hosted total Se concentrations of 1906 μg/L. Unfortunately, attempts to speciate the dissolved Se on an ion chromatograph failed because the elevated concentrations of phosphate and sulfate inhibited detection of selenite and selenate peaks which could not be alleviated with protocol amendment (data not shown). Organisms in filtered WWI appeared healthy throughout the first 15 days of the experiment, but beyond 15 days began to exhibit extreme stress coincident with inhibited growth (Supplementary Figure S2). After 7 days in filtered WWI, *A. alternata* removed only 1% and *Alternaria* sp. removed < 1% of the total Se (Figure 4; Table 2).

**TABLE 2:** Concentrations and rates of Se removal in wastewaters with and without carbon amendments.

**Wastewater**	**Experiment Type**	**Fungal Species**	**Days**	**Se removed (%)**	**Rate (μg/L Se removed per day)**	**Amount removed by 21 days (μg/L)**	**Days to achieve standing water EPA limits (1.5 μg/L)**	**Days to achieve flowing water EPA limits (3.1 μg/L)**	**Solid Se/biomass (μg/mg)**
**Municipal**	Unamended Filtered	*A. alternata*	7	19% ± 3	0.7 ± 0.1	14.6	34.5	32.2	-
		*Alternaria* sp.	7	14% ± 2	0.5 ± 0.1	10.9	46.3	43.3	-
		*A. alternata*	21	15% ± 1	0.2 ± 0.0	3.73	135	126	0.10
		*Alternaria* sp.	21	12% ± 4	0.1 ± 0.0	3.12	162	151	0.04
	Carbohydrate-amended	*A. alternata*	7	25% ± 2	0.9 ± 0.1	18.8	26.8	25.0	0.01
		*Alternaria* sp.	7	24% ± 1	0.9 ± 0.0	18.6	27.1	25.3	0.05
	Glycerin-amended	*A. alternata*	7	12% ± 0	0.4 ± 0.0	8.88	55.2	51.5	0.03
		*Alternaria* sp.	7	9% ± 0	0.3 ± 0.0	6.39	76.8	71.5	0.02
	Unamended Unfiltered	*A. alternata*	7	11% ± 3	0.4 ± 0.1	7.44	60.3	55.8	-
		*Alternaria* sp.	7	15% ± 5	0.5 ± 0.1	10.6	42.2	39.1	-
		*A. alternata*	21	14% ± 2	0.2 ± 0.0	3.19	141	130	0.19
		*Alternaria* sp.	21	19% ± 4	0.2 ± 0.0	4.34	103	95.6	0.29
	Unamended Killed Control	*A. alternata*	7	13% ± 3	0.5 ± 0.1	10.1	50.1	46.7	-
		*Alternaria* sp.	7	15% ± 2	0.5 ± 0.1	11.4	44.4	41.4	-
		*A. alternata*	21	21% ± 2	0.3 ± 0.0	5.33	94.7	88.4	0.05
		*Alternaria* sp.	21	22% ± 2	0.3 ± 0.0	5.60	90.1	84.1	0.03
	Abiotic Controls	*Unamended Filtered*	7	6% ± 1	0.2 ± 0.0	4.23	119	111	-
		*Unamended Filtered*	21	9% ± 1	0.1 ± 0.0	2.24	225	210	0.11
		*Glycerin-amended*	7	4% ± 1	0.2 ± 0.0	1.06	463	431	0.12
**Industrial**	Unamended Filtered	*A. alternata*	7	1% ± 4	2.6 ± 12	54.0	741	740	-
		*Alternaria* sp.	7	0% ± 2	-	-	-	-	-
		*A. alternata*	21	-1% ± 1	-	-	-	-	0.02
		*Alternaria* sp.	21	2% ± 3	2.1 ± 3.1	44.7	895	895	0.02
	Carbohydrate-amended	*A. alternata*	7	8% ± 3	23 ± 9.4	490	90.4	90.3	0.01
		*Alternaria* sp.	7	7% ± 3	20 ± 9.4	430	103	103	0.01
	Glycerin-amended	*A. alternata*	7	0% ± 0	-	-	-	-	0.00
		*Alternaria* sp.	7	-4% ± 2	-	-	-	-	0.00
	Unamended Unfiltered	*A. alternata*	7	-3% ± 2	-	-	-	-	-
		*Alternaria* sp.	7	-10% ± 5	-	-	-	-	-
		*A. alternata*	21	6% ± 2	5.1 ± 1.5	107	356	355	0.02
		*Alternaria* sp.	21	6% ± 1	5.0 ± 0.6	106	360	360	0.02
	Unamended Killed Control	*A. alternata*	7	3% ± 1	7.5 ± 2.6	158	253	253	-
		*Alternaria* sp.	7	1% ± 1	3.7 ± 3.0	78.0	513	512	-
		*A. alternata*	21	9% ± 3	8.3 ± 2.2	175	228	228	0.02
		*Alternaria* sp.	21	10% ± 2	8.9 ± 1.7	186	215	215	0.01
	Abiotic Controls	*Unamended Filtered*	7	0% ± 1	1.0 ± 2.6	21.0	1905	1903	-
		*Unamended Filtered*	21	2% ± 2	2.0 ± 2.4	42.0	952	952	0.02
		*Glycerin-amended*	7	0% ± 2	- -	-	-	-	0.00

Additionally, fungi were grown in unfiltered wastewater to evaluate whether fungal Se removal was feasible in the presence of a complex microbial community, representing a more realistic water treatment scenario. In unfiltered WWI, Se concentrations changed from 1820 μg/L to 1880 and 2008 μg/L Se for *A. alternata* and *Alternaria* sp. respectively, by 7 days (Supplementary Figure S4). Some Se removal eventually was observed between 7 and 21 days – *A. alternata* and *Alternaria* sp. both removed 6% (Table 2).

**FIGURE 3:**
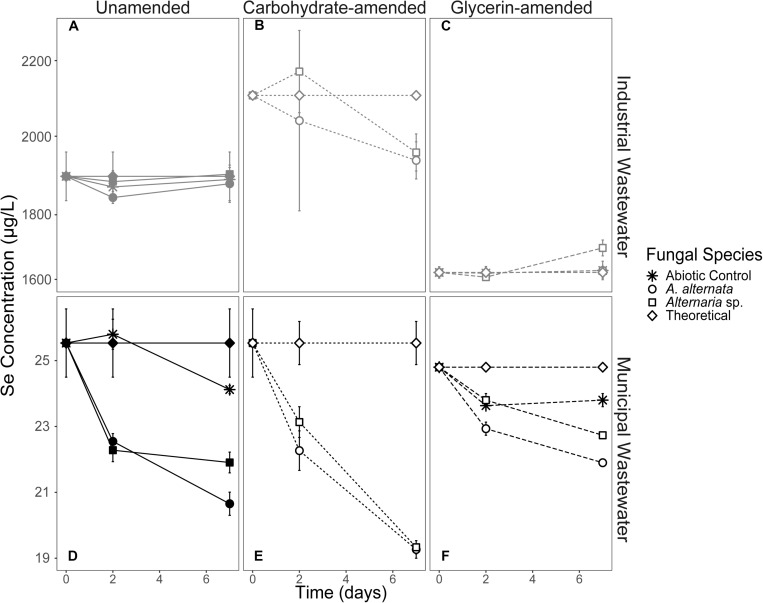
Aqueous Se concentration (μg/L) remaining in solution over time for both WWI (top) and WWM (bottom) unamended **(A,D)**, carbohydrate-amended **(B,E)**, and glycerin-amended **(C,F)** experiments. These fungi remove more Se from solution when grown with a carbohydrate-based carbon amendment compared to any other amendment. Error bars represent the standard deviation calculated from triplicates (unamended wastewater experiments) and duplicates (carbon-amended experiments). For duplicates, data points represent calculated means and error bars represent actual values measured for each duplicate. Symbols without error bars have a standard deviation that is smaller than the symbol size. Theoretical calculations were made using the starting concentration of total Se measured, and assuming that under ideal abiotic conditions, the amount and volume of Se removed during sampling would be replaced when adding fresh media or wastewater back into the flask.

Ethanol-killed controls were also conducted to assess whether abiotic adsorption to fungal biomass contributes to Se removal from solution. During the first 7 days in WWI, total Se decreased approximately 3% in the presence of the ethanol-treated biomasses (Supplementary Figure S5; Table 2). Despite the fungi being inactive, total Se concentrations continued to decrease between 7 and 21 days, resulting in ∼10% total removal. Unexpectedly, more Se was removed from solution via adsorption to dead biomass in the filtered WWI compared to either living fungus in the filtered WWI (Figure 3; Supplementary Figure S4). In abiotic (no biomass) controls established for filtered WWI, 0% of total Se was removed from solution by 7 days, and 2% was removed by 21 days (Table 2).

In the less geochemically complex WWM, the starting total Se concentration was 25 μg/L. Unfortunately, the concentration of Se in the WWM was too close to the instrument’s detection limits (1 mg/L) to obtain accurate values of the two anionic species. The organisms appeared healthier than they did when grown in WWI but were still not as healthy as the AY growth media cultures. In the filtered WWM experiments after 7 days, both fungi removed 14% - 19% Se from solution. In this case, *A. alternata* removed the greater amount of Se compared to *Alternaria* sp. In the unfiltered WWM, a small amount of Se was removed from solution by fungal biomass of both isolates (Supplementary Figure S4), similar to what was observed for the filtered WWM. Concentrations for the unfiltered WWM started at 23 μg/L Se and *A. alternata* removed 11% and *Alternaria* sp. removed 15% after 7 days. Total Se concentrations also dropped by ∼14% in the WWM killed control experiments (Supplementary Figure S5; Table 2). Abiotic controls established here indicate only a small amount (9%) of Se was removed from solution by 21 days (Table 2).

#### Carbon-Amended Wastewater

Filtered WWI amended with carbohydrates started with 2110 μg/L total Se, and after 7 days, *A. alternata* cultures removed 8%, and *Alternaria* sp. removed 7% of the total Se in solution (Figure 3; Table 2). When the two strains were incubated in glycerin-amended WWI, both cultures were unable to remove Se. WWM amended with carbohydrates, on the other hand, improved Se removal relative to the unamended WWM experiments and abiotic removal. With both *A. alternata* and *Alternaria* sp., the starting 25 μg/L Se in solution was reduced by 25% (Figure 3; Table 2). For WWM amended with glycerin, the starting concentration was 25 μg/L Se. *A. alternata* removed 12% and *Alternaria* sp. removed 9% from the solution. Abiotic controls for glycerin-amended cultures indicated a removal of 4% (Table 2).

### Solid-Associated Selenium

#### AY Growth Media Experiments

Biomass collection after the experiments ended allowed us to assess the total amount of Se accumulating in the solid phase as either Se nanoparticles (free or biomass-associated) or biomass-incorporated organoselenium molecules. With this, we determined the potential for hazardous Se-containing waste production during the water treatment process. It is important to note that due to the draw-and-fill sampling scheme and the replenishment of Se-containing media/wastewater, the fungi could have continued to sequester selenium within their biomass (i.e., the solid phase) beyond the ∼630 μg initially available in solution for the higher concentration experiments. With a refill of 50 mL fresh media or wastewater, an additional ∼126 μg Se per sampling point would be added to the experiment. We only report solid-associated Se at experiment end, but aqueous phase Se data is available for days 0–7 (Tables 1, 2), and days 0–21 (Supplementary Table S2). Between day 7 and experiment end, additional Se was sequestered by the fungi, leading to higher solid-phase Se concentrations than what may have been present at day 7.

When cultured in AY growth media, the highest quantity of solid-associated Se was observed after exposure to a starting concentration of 2520 μg/L Se(IV) (Figure 4; Supplementary Table S3). Here, *A. alternata* and *Alternaria* sp. biomass contained 614.6 and 623.4 μg Se, respectively. The amount of solid-associated Se accumulated when the fungal isolates were grown in Se(VI) was substantially lower than for the high Se(IV) growth media. For example, *A. alternata* accumulated only 45.4 μg Se and *Alternaria* sp. accumulated 72.8 μg Se from the aqueous phase in the high Se(VI) experiments, which were statistically different from all other experimental conditions (*p* < 0.05; italicized letters; Figure 4). A similar trend was observed in the low concentration Se experiments (Figure 4; Supplementary Table S3), where solid-associated Se quantities were greater when the fungi were grown in Se(IV) than in Se(VI) (Figure 4; Supplementary Table S3).

The amount of Se associated with fungal biomass was substantially lower when isolates were grown in either carbon-amended growth media, though this may be at least in part due to experiment length. In these carbon treatments, fungi were only exposed to Se for one week as opposed to multiple weeks for the experiments not amended with carbon. *A. alternata* grown with a carbohydrate-based carbon source and high concentration Se(IV) media sequestered 253.7 μg Se, and *Alternaria* sp. sequestered 212.5 μg Se (Figure 4; Supplementary Table S3). When supplemented with a glycerin-based carbon source under these conditions, *A. alternata* and *Alternaria* sp. biomass accumulated 143.7 and 205.9 μg, respectively. Carbohydrate- and glycerin-amended 2000 μg/L Se(IV) media were statistically different from each other, except carbohydrate-amended *Alternaria* sp. Here, concentrations were not statistically different from carbohydrate-amended *A. alternata* or glycerin amended *Alternaria* sp. (*p* < 0.05; italicized letters; Figure 4). Solid-associated Se was substantially lower (<100 μg) when grown in carbon-amended media with ∼2000 μg/L Se(VI) (Figure 4; Supplementary Table S3). While the amount of solid-associated Se measured in carbon-amended, low Se experiments was quite low, these solid Se values are still a substantial portion of the total Se (μg) that was added to these cultures over time. In media containing 25 μg/L Se(IV), *A. alternata* accumulated 0.6 μg Se in the solid phase when amended with the carbohydrate-based carbon and Se(IV) whereas *Alternaria* sp. had 2.0 μg Se. With a glycerin-based carbon source, *A. alternata* sequestered more Se (3.8 μg) but *Alternaria* sp. accumulated less (1.3 μg Se) than when grown with the carbohydrate-based source. The biomass sequestered minimal Se when amended with the low Se(VI) concentration. Here, when *A. alternata* and *Alternaria* sp. were amended with carbohydrates, only 0.15 μg and 0.32 μg Se were associated with the solid phase (Figure 4; Supplementary Table S3). These levels were even lower when glycerin was used 0.05 and 0.24 μg Se was accumulated by *A. alternata* and *Alternaria* sp., respectively (Figure 4; Supplementary Table S3). For most conditions, 25 Se(IV and VI) experiments were not statistically different (italicized letters; Figure 4).

#### Wastewater Experiments

**FIGURE 5:**
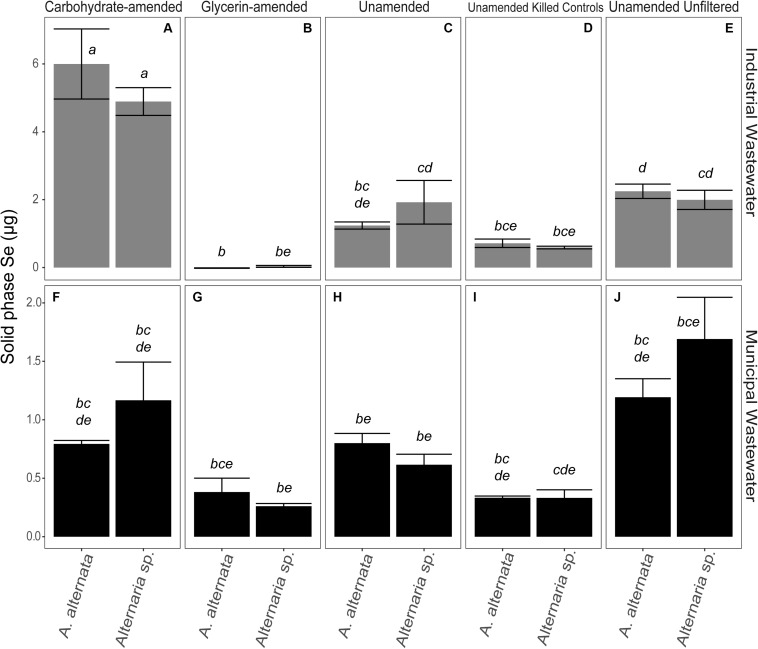
Total Se associated with the solid phase at experiment end for carbohydrate-amended **(A,F)** and glycerin-amended wastewaters **(B,G)**, unamended filtered wastewaters **(C,H)**, killed controls **(D,I)**, and unfiltered wastewaters **(E,J)**. Note that biomass in the experiments without carbon amendments grew in the presence of Se for 21 days, and carbon-amended experiments were incubated for only 7 days. There is a considerable transition of Se into the solid phase for unfiltered wastewater over time. Error bars represent the standard deviation calculated from triplicates (unamended wastewater experiments) and duplicates (carbon-amended experiments). Statistically different solid-associated Se accumulations (*p* < 0.05) among different treatments are indicated by different lowercase letters.

In the filtered WWI experiments (∼2000 μg/L Se), 1.24 and 1.92 μg Se were associated with the solid phase for *A. alternata* and *Alternaria* sp., respectively. This represents ∼0.5% of the solid-accumulated Se when these fungi were grown in growth media with similar Se (as selenite) concentrations. The parallel experiments with lower Se concentrations in WWM yielded 0.8 and 0.6 μg Se for the respective fungi (Figure 5; Supplementary Table S3). Interestingly, the solid-associated Se produced in the unfiltered wastewater was substantially higher than that produced in the parallel filtered wastewater experiments. For example, *A. alternata* and *Alternaria* sp. biomass in the unfiltered WWI contained 2.2 and 2.0 μg Se in the solid phase, respectively (Figure 5; Supplementary Table S3). When WWM was not filtered, 1.2 and 1.7 μg Se were biomass-associated.

The amount of Se accumulated by the biomass was clearly influenced by additional carbon amendments, though the magnitude and direction of change depended on the carbon source. When carbohydrates were added to filtered WWI, *A. alternata* accumulated 6.0 μg solid-associated Se and *Alternaria* sp. produced 4.9 μg solid-associated Se which was a statistically significant change for both organisms (Figure 5). Conversely, when glycerin was added as a carbon source to the WWI, no selenium was measured in the solid phase. When filtered WWM was amended with carbohydrates, solid-associated Se concentrations stayed the same with *A. alternata* (0.8 μg Se) relative to the unamended WWM experiments but increased slightly with *Alternaria* sp. (1.2 μg Se). With glycerin, Se levels were lower than the unamended and carbohydrate-supplemented experiments – 0.4 and 0.3 μg Se were associated with the solid phase for the respective organisms (Figure 5; Supplementary Table S3), but these differences are not statistically significant.

Dead fungal biomass also accumulated some Se, although these levels were less than when the fungi were alive and able to actively transform Se compounds. The fungi in the WWI killed controls scavenged 0.7 and 0.6 μg Se to the solid phase for *A. alternata* and *Alternaria* sp. In killed control cultures for WWM, 0.3 μg Se was in the solid phase for both organisms. Abiotic controls for WWI and WWM measured ∼ 0.6 and 0.2 μg associated with the solid phase, respectively (Supplementary Table S3).

### Fungal Growth

**FIGURE 6:**
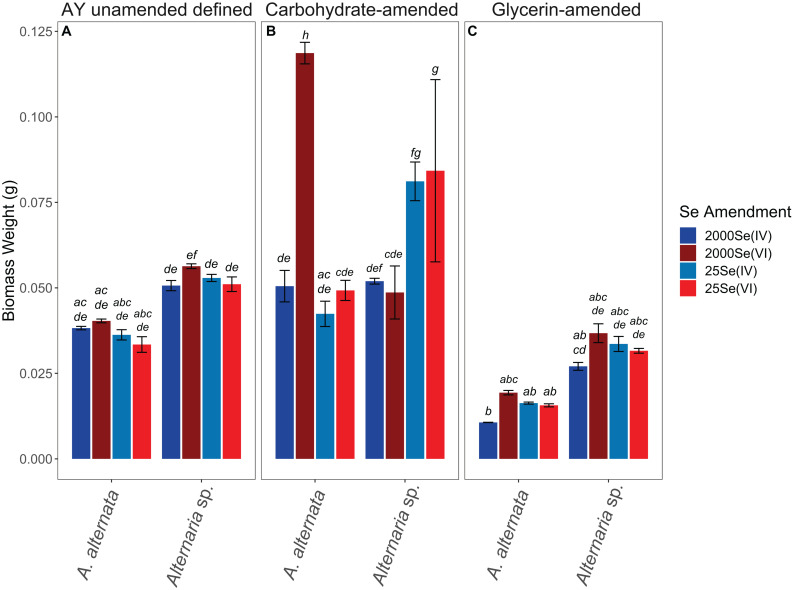
Biomass weights for all positive control experiments. Note that AY growth media **(A)** ran for 21 days whereas carbon-amended experiments **(B,C)** ran for only 7 days before biomass was collected. Biomass weight at experiment onset was 15 and 27 mg for *A. alternata* and *Alternaria* sp. respectively. Error bars represent the standard deviation calculated from triplicates (AY growth media experiments) and duplicates (carbon-amended experiments). Filter weights have been subtracted from values of biomass weight. Statistically different biomass weights (*p* < 0.05) among different treatments are indicated by different lowercase letters.

In AY growth media experiments, the measured values for *A. alternata* and *Alternaria* sp. supplemented with Se(IV), regardless of concentration, increased by 22 mg and 25 mg at the end of the experiment, respectively, relative to the biomasses of 15 and 27 mg at the start of the experiment (Figure 6; Supplementary Table S4). Se(VI)-amended cultures of *A. alternata* increased by 18 mg and 13 mg for low and high Se concentrations, respectively. Se(VI)-amended cultures for *Alternaria* sp. increased by 36 mg (low Se conditions) and 29 mg (high Se conditions).

The biomasses produced by the end of the carbon-amended growth media experiments were more variable than the consistent trends observed in the AY growth media experiments. Cultures grown with the glycerin-based carbon source did not grow as well as the other experiment cultures. With glycerin-amendment, *A. alternata* exposed to 2000 μg/L Se weighed less than the estimated starting biomass [decreased by 4 mg and 8 mg for Se(IV and VI), respectively]. *Alternaria* sp. biomass increased, however, by 12 and 10 mg for Se(IV and VI), respectively. When grown in the presence of a lower concentration of selenite (25 μg/L) and glycerin, *A. alternata* biomass increased by 1 mg regardless of Se oxyanion present, whereas *Alternaria* sp. increased by ∼6 mg. Conversely, the biomass achieved at the end of the carbohydrate-amended experiments was, for most experimental conditions, substantially higher but also more variable both between treatments and between replicates than the parallel AY growth media experiments (Figure 6; Supplementary Table S4). Here, both isolates inoculated with high Se concentrations grew to ∼36 and ∼24 mg respectively, with the exception of *A. alternata* exposed to Se(VI). This culture unexpectedly grew to approximately double that of the others (104 mg). This growth was also evident by the excessive volume of wet fungal mycelia captured during filtration of the experiments. *A. alternata* exposed to low Se concentrations increased by ∼31 mg, but *Alternaria* sp. grew to ∼56 mg in the presence of either selenite or selenate. Tukey’s HSD *post hoc* test indicated that almost all AY media and carbohydrate-amended media were not statistically different from each other but were statistically different from some of the glycerin-amended conditions (italicized letters; Figure 6).

**FIGURE 7:**
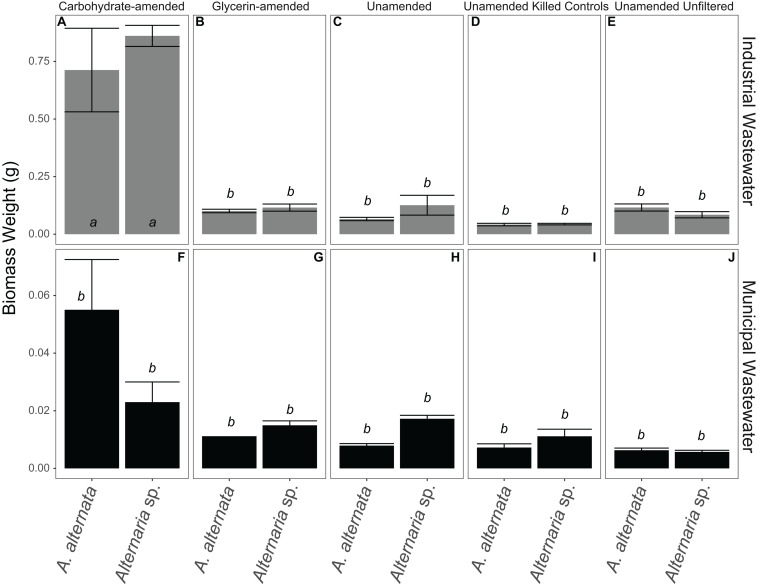
Biomass weights for all WWI **(A–E)** and WWM **(F–J)** experiments. Note that carbon-amended experiments ran for only 7 days, whereas unamended wastewaters ran for 21 days before biomass was collected. Error bars represent the standard deviation calculated from triplicates (unamended wastewater experiments) and duplicates (carbon-amended experiments). Statistically different biomass weights (*p* < 0.05) among different treatments are indicated by different lowercase letters.

Biomass varied in the unamended filtered and unfiltered WWI experiments. In the experiments where WWI was unfiltered – *A. alternata* biomass weight increased by 101 mg whereas *Alternaria* sp. grew less than in the filtered experiments (58 mg). The biggest and statistically significant improvement in Se removal was when carbohydrates and nutrients were added to WWI (*p* < 0.05; italicized letters; Figure 7). *A. alternata* increased to 698 mg and *Alternaria* sp. to 834 mg. Comparatively, adding the glycerin-based carbon source and additional nutrients resulted in less overall growth; the biomass of *A. alternata* increased from the starting biomass weight by 86 mg and *Alternaria* sp. increased by 89 mg. However, it is important to note that the WWI was extremely difficult to filter at each sampling point, suggesting precipitation of dissolved components or some interaction of dissolved components with living fungi in this water. This may have artificially increased the measured biomass, skewing our interpretation of the fungal growth in WWI (Figure 7; Supplementary Tables S3, S4). In the unamended filtered WWM, biomass decreased by 7 mg for *A. alternata* and 10 mg for *Alternaria* sp. (Figure 7; Supplementary Table S4) by experiment end. In unfiltered WWM at experiment end, *A. alternata* and *Alternaria* sp. decreased by 9 and 21 mg. This small number was reflected in the visual appearance of the fungal mycelia, which appeared black, shriveled, and flaky. Although not as significant of a change as in experiments with WWI, WWM produced greater biomass (relative to unamended experiments) when supplemented with carbon, particularly when amended with carbohydrates. *A. alternata* increased by 40 mg but *Alternaria* sp. decreased by 5 mg, respectively. When exposed to ethanol, *A. alternata* increased by ∼28 mg and *Alternaria* sp. decreased by ∼18 mg at the end of the WWI and WWM experiments indicating solid accumulation onto dead biomass. Abiotic controls all weighed less than 3 mg, except for those exposed to filtered WWI. Abiotic filtered WWI weighed 52 mg and abiotic filtered WWI with glycerin weighed 56 mg (Supplementary Table S4). Any detectable weights for the abiotic controls are presumably due to accumulation of solid particulates.

## Discussion

### Selenium Remediation Opportunities

The USEPA recommends total Se concentrations in static water are <1.5 mg/L and <3.1 mg/L in flowing water (US EPA, 2016). Although the USEPA regulates total selenium concentrations in the environment, the predominant forms of selenium present in surface waters are the oxyanions, selenate [Se(VI); SeO_4_^2–^] and selenite [Se(IV); SeO_3_^2–^]. The reduction of selenate and selenite to insoluble elemental Se(0) or volatile Se(-II) forms reduces the bioavailability and most hazards associated with Se in the environment (Lenz and Lens, 2009; Nancharaiah and Lens, 2015b; Chan et al., 2018). As such, a more appropriate goal is to remove Se from the aqueous form and convert it to Se(0) or Se(-II). In water treatment applications, elemental Se(0) is ideal, as it can be physically separated from treated wastewaters (Lenz et al., 2008; Winkel et al., 2012; Nancharaiah and Lens, 2015a). However, the preferred Se reduction product could depend on the environmental conditions, the treatment strategy (e.g., closed or open system), and the Se form present.

A handful of studies have discussed fungi capable of Se transformations (Karlson and Frankenberger, 1989; Gharieb et al., 1995; Rosenfeld et al., 2017; Kieliszek, 2018), yet there is limited knowledge about their ability to remove Se from wastewater. Further, a limited number of studies have compared selenate and selenite reduction by the same organism (Tomei et al., 1992; Gharieb et al., 1995; Rosenfeld et al., 2017, 2020; Tan et al., 2018). This study is one of the first to assess an organisms’ potential for both selenate and selenite reduction in an applied water treatment strategy for complex Se-laden wastewater. Previous research with these fungi has not addressed time dynamics associated with Se reduction processes, especially those relevant to water treatment timeframes. A strength of this work is that we assess Se removal at several time points over multiple weeks. For the first time, we examined the potential for two filamentous Ascomycete fungi to survive, grow, and remove Se from industrial and municipal wastewater sources. *A. alternata*’s previously described ability to convert Se to both solid and volatile forms (Rosenfeld et al., 2017) makes it a prime candidate for bioremediation work due to this flexibility.

### Assessing and Improving the Se Mycoremediation Potential

Through this study, we showed that these two strains of filamentous fungi have the capacity to remove large quantities of aqueous selenium from solution under oxic conditions. In all growth media experiments, both fungi removed Se from solution, regardless of oxidation state given (Figure 2; Table 1). From a previous study of Se reducing fungi (Rosenfeld et al., 2017), we expected that both *A. alternata* and *Alternaria* sp. would perform better when exposed to selenite compared to selenate. As expected, we found that both fungal isolates indeed remove larger quantities of selenite from solution, incorporate more selenite than selenate into the solid phase, and facilitate higher rates of selenite removal than selenate removal (Table 1). The rate of Se removal in AY growth media supplemented with a high concentration of Se(IV) was notable as demonstrating high potential for bioremediation. In this growth media without an industry-approved carbon amendment, *A. alternata* and *Alternaria* sp. respectively were able to remove a substantial amount (∼75%) of the initial 2000 μg/L Se(IV) in solution by 7 days (Table 1), at a rate of ∼270 μg/L Se per day. At this rate, it would take less than 10 days to achieve standing (1.5 μg/L Se) and flowing (3.1 μg/L Se) water USEPA limits (Table 1). Of note, however, Se removal rates decreased over time, potentially due to progressive nutrient limitation as the fungi consumed the media components. An exchange of biomass or continual carbon or nutrient addition within a bioreactor could eliminate this issue.

The fungi also removed Se(VI) from solution, though to a lesser extent than Se(IV). The rate of removal in the AY growth media experiments was closer to 30 μg/L Se per day with a high Se concentration, and 0.86 μg/L Se per day with a low Se(VI) concentration. Based on these rates, it would take a calculated 65 days (high concentration selenate) and 28 days (low concentration selenate) to reach USEPA limits for either standing or flowing water. As the genetic mechanism(s) of fungal Se reduction are currently unknown, the reduction pathways are not yet fully understood. Some toxicology studies suggest that Se(IV) is more toxic than Se(VI) in plants (e.g., Kolbert et al., 2018; Molnár et al., 2018), which could be the case for fungi as well. As such, the mechanisms for Se(IV) detoxification may be very effective and efficient, particularly at higher Se loadings. The Se(IV/VI) reduction rates and biomass weights measured here in AY growth media indicated that although the biomass was approximately the same weight at the end of both Se(IV and VI) experiments, more Se(IV) was removed and incorporated into the solid phase for Se(IV) experiments (Table 1; Figures 4, 6; Supplementary Tables S2–S4). Solid phase Se was likely generated through production of biomass-affiliated Se(0) nanoparticles as well as production of organoselenium compounds within the biomass (Rosenfeld et al., 2020). Future research should be directed at resolving the fungal metabolic pathways involved in both selenate and selenite reduction to provide more targeted approaches at improving the bioremediation strategy.

In an effort to boost Se removal rates and efficiencies, we provided carbon supplements that we suspected were lacking in the wastewater and limiting fungal growth and activity. The supplements were chosen based on their ability to be used in a functional bioreactor designed for industry and municipal wastewater treatment. Largely, the carbohydrate-based carbon supplements outperformed the glycerin-based supplements suggesting that glycerin is not a preferred carbon source for these fungi, at least at the concentrations provided. While the carbohydrate-amended media cultures had more variability, potentially due to the complexity of this proprietary carbon source, they all had higher biomass weights than the glycerin-amended cultures (Figure 6).

### Selenium Mycoremediation Performance in Wastewater

While the fungi in the growth media experiments removed Se efficiently under all the conditions (high vs. low Se concentration, AY growth media vs. carbon-amended) tested, fungi grown in Se-containing WWI and WWM struggled by comparison. Selenium (undetermined speciation) removal was particularly ineffective in the WWI. All wastewaters host varying levels of chemical complexity, and this can have a substantial impact on the growth, activity, and viability of organisms in the fluid. It is possible that the wastewaters here could have had multiple forms of Se in solution present as selenate, selenite, or even as colloidally suspended solid organo- or elemental-Se forms. Recent work by Rosenfeld et al. (2020) revealed the influence of Mn on the Se cycle, so it is possible that more cryptic interactions between Se and wastewater components were occurring and influencing the form and concentration of Se species. Although the wastewater was filtered (<0.2 μm) prior to initial fungal inoculation, the WWI was extremely difficult to filter at each experimental sampling point, suggesting precipitation of dissolved components or some interaction of dissolved components with living fungi was an ongoing phenomenon in this water. This may have artificially increased the measured biomass weight, skewing our interpretation of the biomass accumulation in WWI (Figure 7; Supplementary Tables S3, S4). The filtered WWI also appeared to increase in Se concentration between 7 and 21 days when grown with *A. alternata* (Table 2). The fungi in this wastewater displayed visual evidence of extreme toxicity including dark pigmentation and shriveled biomass by 21 days (Supplementary Figure S2). It is suspected that some biomass-associated Se was released after initial sequestration upon cell death and lysis.

The unfiltered wastewaters included additional complexity, from both a chemical and biological perspective. While competition and interaction between bacterial and fungal organisms was presumed in the unfiltered wastewaters, when no carbon additions were made, both fungi removed about the same amount of Se from solution in the unfiltered wastewaters as they did in the filtered wastewater experiments (Table 2). In the unfiltered WWI experiments, it appeared Se actually accumulated in solution by 7 days, but by 21 days percent removal increased to 6% and exceeded that of the filtered samples (Table 2). This extra removal in unfiltered wastewater may be, at least in part, due to removal by other microbes present or various abiotic processes. As discussed above, it was also probable that some of the fungal cells died in this toxic water and re-released some sequestered Se back to solution.

The WWM on the other hand, hosted lower total Se and total dissolved solids concentrations, and minimal signs of toxicity were observed in the filtered water. In the filtered WWM, the fungi were successful in removing ∼17% of the dissolved Se from the wastewater within 7 days (Table 2; Figure 3). In unfiltered WWM, approximately the same proportion was removed within 7 days, indicating that the fungi are capable of performing this reduction even in a mixed microbial community, which is a more realistic wastewater treatment scenario (Supplementary Figure S4). However, despite moderate Se removal, the WWM still may have lacked a sufficient carbon source for the organisms to thrive.

Solid-associated Se in both unfiltered wastewaters was higher than in the respective filtered wastewaters (Figure 5; Supplementary Table S3), which may have been due to solid Se already present in the unfiltered water, or resident microbes reducing Se in wastewater before the fungi were added. As the fungi expressed toxicity and decreased viability in both unfiltered wastewaters between 7–21 days (Supplementary Table S4), it is unlikely that the fungi in the unfiltered waters outperformed the fungi in the filtered waters with respect to Se transformation from the dissolved phase to the solid phase. Rather, it is more likely that a portion of Se was present as a solid in the initial wastewater. As the Se concentrations were determined via ICP-OES or ICP-MS and were unable to be speciated, as discussed above, these measurements likely included both solid phase and dissolved Se present in the initial unfiltered wastewater. Perhaps surprisingly, measurable Se removal occurred via killed fungal biomass that rivaled removal rates in the respective unamended wastewater experiments (e.g., WWI experiments) by living fungi (Table 2). This suggests that fungal-mediated removal was at least partially due to abiotic processes. As abiotic controls generally removed less Se than unamended and killed control experiments, adsorption to biomass was likely the primary mechanism here. For example, sulfides and thiols can have a large impact on Se oxidation states (Sharma et al., 2015). It is also possible that the fungi may have survived incubation in 100% ethanol for two days, though previous lab experiments have demonstrated that 2 days was sufficient to prevent fungal growth and there was no observable fungal growth in the wastewaters following ethanol treatment. It is also possible that even after ethanol treatment, intracellular proteins capable of reducing Se oxyanions persisted. However, as the ethanol treatment is expected to have inhibited all active cellular uptake, this is not expected to be a viable pathway for Se removal. It is also feasible that ethanol treatment resulted in a release of Se-reducing intracellular proteins to solution, which could contribute to the observed Se removal by the ethanol-treated biomass. Since the Se reduction mechanisms remain unresolved, this interpretation is still speculative and requires further investigation.

### Carbon Source Impacts Fungal Selenium Removal

More Se was removed from WWM with a carbohydrate-based carbon supplement compared to glycerin or unamended experiments. When the fungi were grown in WWM without a carbon amendment, Se remaining in solution dropped by ∼17% for both fungal species within 7 days (Table 2). During these 7 days, the rate of removal was 0.7 and 0.5 μg/L Se per day for *A. alternata* and *Alternaria* sp. respectively. At this rate, the Se remaining in solution would drop below the USEPA Lentic Criterion of 1.5 μg/L Se in standing water by either 35 or 46 days for *A. alternata* and *Alternaria* sp., respectively. When cultures in WWM were supplemented with carbohydrates, after 7 days, the fungal-mediated removal rate increased to ∼0.9 μg/L Se per day. At this rate, the Se remaining in solution would drop below USEPA Lentic and Lotic Criteria of 1.5 μg/L Se in standing water (lentic) by 27 days, and 3.1 μg/L Se in flowing (lotic) water by 25 days (Table 2). Although biomass in the unamended WWI grew 3x longer, higher concentrations of solid-associated Se were observed in carbohydrate-amended waters (Figure 5). In both wastewaters, *A. alternata* and *Alternaria* sp. grown with a carbohydrate-based carbon supplement produced considerably more biomass (∼75 mg in WWI, ∼40 mg WWM) than any other wastewater treatment (Figure 7). These similarities highlight the effectiveness of carbohydrates in fungal growth and the process of removing Se from solution. Due to the lack of both fungal growth and Se removal from solution in these experiments overall, it is apparent that the fungi were generally less tolerant of this glycerin-based carbon amendment. To more accurately understand the genetic mechanisms involved with fungal Se removal and in turn help improve any fungal bioremediation strategy, future studies should focus on the effect of carbon sources with a definite composition and concentration, such as glucose, acetate, or other environmentally relevant carbon sources.

### Practical Considerations

Assessment of this fungal-based selenium removal process as a future water treatment strategy highlights many potential strengths and possibilities. For example, as the process is aerobic, there is no need to remove dissolved oxygen from the contaminated water and it could be conducted directly in a pond or lagoon with relatively low maintenance. Also, the fungi are capable of reducing elevated concentrations of both Se(VI and IV) directly to reduced forms of Se [e.g., solid Se(0) and, likely, volatile Se(-II)] without the buildup of any Se(IV), as has been observed in certain instances (e.g., Fujita et al., 1997).

This fungal water treatment strategy appears to be most readily applicable to Se(IV)-bearing wastewaters or to polishing effluent from systems where Se(IV) has accumulated. While the fungi were able to remove small quantities of Se(VI) from solution, they clearly were more efficient in Se(IV) removal. On their own, the fungi show immense potential as an effective Se(IV) treatment strategy. Employing a bacterial Se(VI) reduction step before fungal Se(IV) removal could facilitate rapid Se(IV/VI) treatment. The capacity of the fungi in this study to reduce Se(IV) to less toxic forms is a logical combination with bacterial Se(VI) reduction to completely reduce and remove Se(VI) in a stepwise fashion. A two-step strategy such as this would ensure Se removal, regardless of oxyanion present. Further because the addition of supplemental carbon (as carbohydrates) to the fungal reactor aids in fungal growth and Se removal (Table 2), leftover carbon from an upstream bacterial process could enhance this water treatment strategy. If more carbohydrates are required, supplementing with a readily available and industry-approved carbon source is possible.

In order to optimize this fungal Se removal strategy, future work should focus on increasing the rate of Se removal over shorter time frames. From this work, it is evident that there may be an optimum Se load for obtaining high removal efficiency. Fungi in WWI had higher removal rates (ranging from 2.1 – 23 μg/L Se per day) than WWM (ranging from 0.1 – 0.9 μg/L Se per day; Table 2), despite inefficiencies potentially related to Se form and other wastewater components. To this end, future work should examine the optimal Se load for these fungi and use that as a specification when identifying waters applicable to this treatment strategy. However, the concentration at which other metals and total dissolved solids are toxic to the fungi should also be examined. As high dissolved metal concentrations likely hindered the growth and Se-removal efficiency of the fungi in our experiments, it would be ideal to remove other metals prior to Se removal in a stepwise water treatment strategy. Also, as an engineered system would include controls to manage the ratio of Se to biomass and the biomass age in the reactor (e.g., solids separation and recycling of biomass to the reactor, respectively), a test to assess the effect of different biomass concentrations or ages on Se removal efficiency would be beneficial. When scaling up to a pilot or full-scale operation in a water treatment setting, a large, open fungal reactor would be most effective if located after initial screening, coagulation and flocculation, sedimentation, and filtration steps, but before any final disinfection steps. Placing the fungal Se reactor after filtration would allow for the removal of pathogenic organisms and potential fungal competitors. A second filtration step would be necessary to remove fungal cells from the treated water.

### Knowing Se Speciation Is Essential for Effective Treatment of Se-Contaminated Sites

While current policies require that total Se levels remain below 1.5 μg/L for standing water and 3.1 μg/L for flowing water, there is no regulation about the specific form of aqueous Se present and thus Se speciation is usually not determined in most wastewater streams. As shown through the results of this current study, Se removal efficiency is highly dependent on Se oxidation state, form, and starting concentration in solution. Some organisms are better equipped to remove excess selenate from solution (e.g., Butler et al., 2012), while others, like the fungi here, more efficiently remove selenite. By measuring and understanding the initial form(s) of Se present in the wastewaters, a more targeted and efficient water treatment strategy can be developed.

As described in the results section above, Se speciation could not be determined in the wastewaters. After comparison to the positive controls, we hypothesize that selenate is likely the dominant form of Se in both wastewaters. The fungal Se removal over time in 2000 μg/L and 25 μg/L Se(VI) in WWI plots is consistent with the patterns of removal in AY media amended with Se(VI) (Figures 2, 3). While Se was not efficiently removed from the WWI (∼90 days to reach EPA limit), these fungi do remove Se from the WWM amended with carbohydrates and exhibit promise as an effective water treatment strategy in this setting. With the addition of the carbohydrate-based amendment, fungal growth and Se removal was enhanced and could achieve EPA limits by ∼25 days. Applications where Se is present predominantly as Se(IV) may be more amenable to this method as these fungi and several others (e.g., Rosenfeld et al., 2017) more efficiently reduce selenite compared to selenate. Future studies should examine the applicability of fungal reduction of selenite-contaminated wastewaters.

### Potential for Se-Leaching From Biomass and Recycling of Sequestered Se

While the goal of any water treatment strategy is to reuse or recycle the contaminant of concern if possible, some products may require disposal. To determine whether the amount of solid-associated Se is likely to be classified as hazardous waste, a leachate calculation was performed. Rather than conduct the USEPA’s Toxicity Characteristic Leaching Procedure (TCLP; US EPA, 1992; Schwartz et al., 2018) in the laboratory due to the low levels of Se associated with the biomass, the Rule of 20 calculation (Minnesota Pollution Control Agency, 2011) was implemented. The Rule of 20 states that 1 mg/L in TCLP extract defines a hazardous waste. As the TCLP test involves a 20-fold dilution of the leachate, 1 mg/L must be multiplied by 20 for accurate comparison. Therefore, a likely hazardous waste would exceed 20 mg/kg. The highest Se concentration measured in the solid phase for wastewater samples was ∼18.44 mg/kg (0.0026 mg Se, 141 mg fungal biomass; WWI unfiltered, *A. alternata*). Thus, the total Se sequestered on and within the fungal biomass under these conditions is not likely to be considered a hazardous waste. However, the highest measured concentration of total solid-associated Se from the positive non-wastewater controls was 12,138.32 mg/kg (0.6494 mg Se, 53.5 mg biomass; 2000 μg/L Se(IV), *Alternaria* sp.). This experiment performed much more efficiently than the actual wastewater-containing experiments and far exceeded the 20 mg/kg threshold. In this case, TCLP testing would be required to confirm the hazardous waste status. As the positive control cultures were not undergoing active remediation, this was not an issue for the current study but should be considered when optimizing this process for environmental settings.

Previous studies have shown that these macroscopic fungi effectively generate and sequester copious Se nanoparticles (Rosenfeld et al., 2017). This is also evident from the results from the high Se(IV) conditions in this study. Se has great economic and ecological value, so the mycoremediation process could have further benefit in downstream applications and contribute to a circular economy where the Se nanoparticles are recycled and repurposed for their maximum value. For example, these nanoparticles could be separated from the fungal biomass and reused for biotechnological or medical applications (Wang et al., 2015; Nie et al., 2016; Fang et al., 2018) rather than classified as hazardous waste and sent to a landfill. The accumulation of organically bound Se (e.g., selenocysteine and selenomethionine) compounds and Se(0) nanoparticles by these fungi could also produce a feedstock supplement (Skalickova et al., 2017) which is commonly provided to livestock as selenized yeast (Schrauzer, 2006).

## Conclusion

Our study showed that under optimal conditions, filamentous Ascomycete fungi can remove large quantities of both selenite [Se(IV)] and selenate [Se(VI)] to levels that meet current USEPA recommendations for aquatic life in as little as 9 days under the conditions studied. The removal rates and total Se removed was higher for Se(IV) than Se(VI) under the conditions tested. Regardless, both organisms demonstrated considerable potential for use within a water treatment approach. We find that given a combination of complex wastewater chemistry and Se oxidation state in solution, the fungi are still able to partially remove Se from WWM. The extremely high concentration and combination of metals, sulfate, and other components in the WWI likely played a major role in the diminished efficiency of fungal Se removal. Further, as the addition of carbohydrates aided in the removal of Se from solution, we note that the form of organic carbon present can impact the amount of Se removed from solution. While the fungal metabolic pathways for Se(IV or VI) reduction are not yet known, future studies should focus on the identification of these pathways, as water treatment efforts could be optimized for carbon source and nutrient demands as well as for certain scenarios where either elemental Se(0) or volatile Se(-II) production is desirable. We show that before any water treatment strategy is implemented to address Se contamination, it is imperative to understand not just total Se in the water as required by the USEPA, but also the phase(s) of Se in solution if possible, as some organisms are better equipped to remove certain Se forms than others. As dissolved oxygen needs to be increased in treated water before discharge, removal of any residual Se(IV) by these fungi has potential to also be a polishing step to an anaerobic Se(VI) treatment process. Finally, rather than disposing of the sequestered Se as hazardous waste, we suggest that a higher priority should be placed on recycling and repurposing the nanoparticulate Se in biotechnological applications when possible.

## Data Availability Statement

The sequence for *Alternaria* sp. CMED5rs1aP4 generated for this study can be found in GenBank under the accession number SUB7400600 (MT444989).

## Author Contributions

CR, MS, TD, and CS created and designed the study. KS, CR, and MS performed laboratory experiment set-up, conducted experiments, and analyzed data. TD and KW-J supplied the wastewaters and assisted with data interpretation. MS and CR created the figures. MS wrote the manuscript and created final figures and tables with input, critical discussion, and edits from CR, TD, KS, KW-J, and CS.

## Conflict of Interest

TD and KW-J were employed by Geosyntec Consultants.

The remaining authors declare that the research was conducted in the absence of any commercial or financial relationships that could be construed as a potential conflict of interest.
