# Supplementary material for: Fungal Bioremediation of Selenium-Contaminated Industrial and Municipal Wastewaters
Source: Front Microbiol. 2020 Sep 8;11:2105. doi: 10.3389/fmicb.2020.02105 (PMC7507899; doi:10.3389/fmicb.2020.02105)
Supplement: Supplementary file 1 [file Data_Sheet_1.PDF]

Supplementary Material

1 Supplementary Data

Chemical Oxygen Demand (COD) calculations for AY media and MicroC products in media and wastewaters

1. Calculate AY medium COD content.

   AY media = 0.24g sodium acetate / 980 mL H_2O
   Sodium acetate COD = 1.07 g COD/g Acetate
   (0.24 g sodium acetate) * ((60 g acetate) / (82 g sodium acetate)) * ((1.07g COD) / (1g acetate)) = 0.188 g COD

   AY media = 0.15g yeast extract / 980 mL H_2O
   Yeast extract COD (assuming glutamic acid) = 0.98 g COD/g yeast extract
   ((0.15g yeast extract) * ((0.98 g COD) / (1g yeast extract))) = 0.147 g COD
   (0.188 g COD acetate) + (0.147 g COD yeast extract) = 0.334 g COD / 980 mL H_2O
   ((0.334 g COD) / (0.980 L)) = 0.342 g COD/L or **342 mg/L COD**.

2. Calculate equivalent dose of MicroC 4000

   COD content = 167,500 mg/1L MicroC
   Density = S.G. = 1.235 * 1000 g water/L = 1,235 g/L
   (342 mg/L COD AY media) * (1L MicroC 4000 / 167,500 mg COD) =
   0.0207 mL MicroC /L

3. Calculate equivalent dose of MicroC 2000

   (342 mg/L COD AY media) * (1L MicroC 2000 / 1,100,000 mg COD) = 3.11E-4 L MicroC /L or **0.31 mL MicroC/L water**

4. Estimate nitrogen content of AY media

   * assume yeast extract source as glutamic acid
   (0.15 g yeast extract)* ((0.07g N) / (1g yeast extract)) = ((0.0105 g N) / (0.98 L)) =
   10 mg N / L

5. Estimate phosphorus content of AY media

   Assume 0.1% P in yeast extract (nutritional yeast)
   (0.15 g Yeast extract) * ((0.001 g P) / (1g yeast extract)) = **1.5 mg P/L**
   Compare P to content of wastewaters.

6. Check Ratios.

   Rough C estimate
   342 mg COD / L * 12g C/32g O_2 = 128 mg C /L
   128 mg C : 10 mg N : 1.5 mg P

85 mg C : 7 mg N : 1.0 mg P
100 : 10 : 1 is typical for bacterial wastewater treatment, so this ratio of C:N:P is not far off for testing fungal wastewater treatment.

2 Supplementary Figures and Tables

2.1 Supplementary Figures

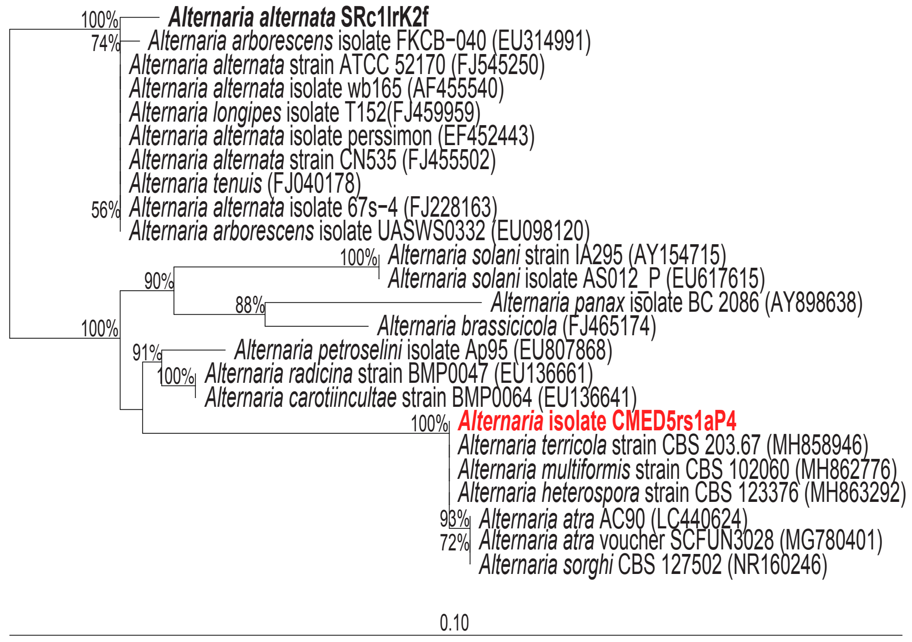

Supplementary Figure 1. Phylogenetic Tree for fungal isolate *Alternaria* sp. CMED5rs1aP4 (red), a previously isolated (Santelli et al., 2010) Se-transforming *Alternaria alternata* strain SRc1lrK2f also in this study (bold), and closely related organisms.

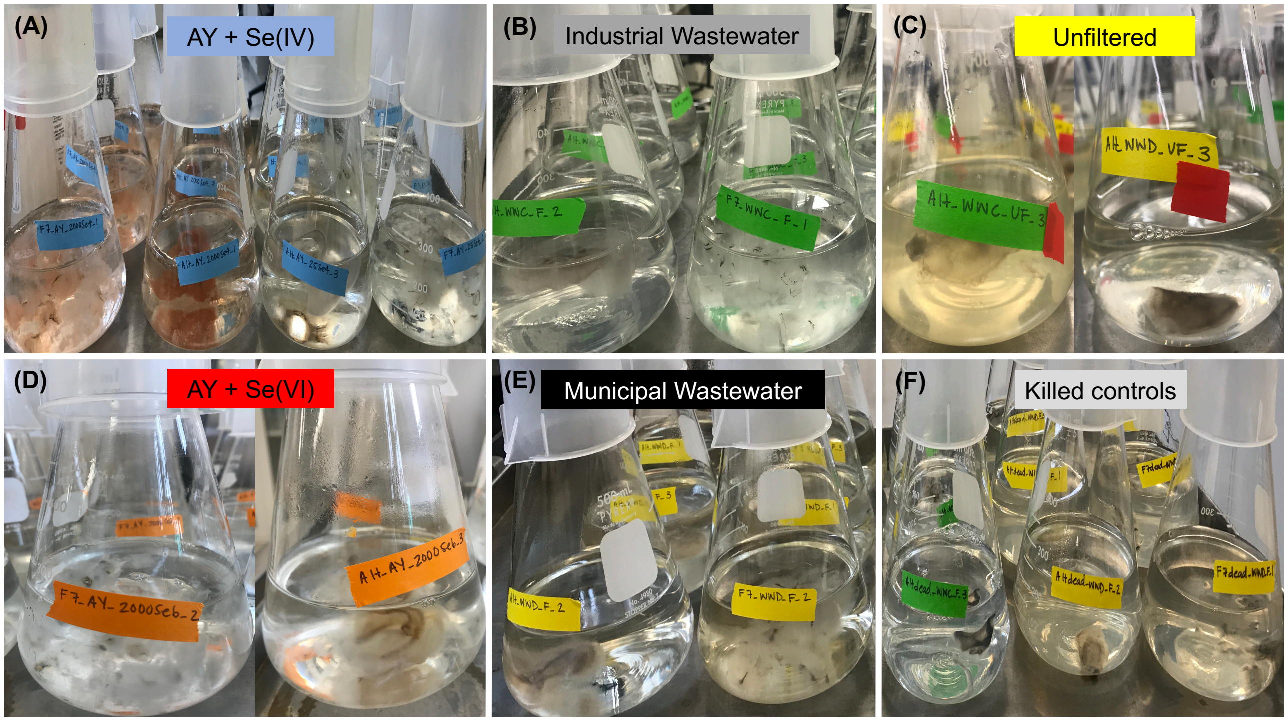

Supplementary Figure 2. Images of fungal biomass grown in AY growth media with selenite or selenate (A, D), WWI (B), WWM (E), unfiltered wastewaters (C), and killed controls (F) at day 7 of the experiments. Flasks in the background of each panel are replicates of flasks in the foreground. Red-pink color in AY growth media + Se(IV) fungal biomass indicates the presence of biogenic nanoparticulate Se(0). Note biomass in unfiltered samples (top right) appears similar to killed controls (bottom right). The dark-colored biomass in many of the experiments is due to the presence of a pigment, likely melanin.

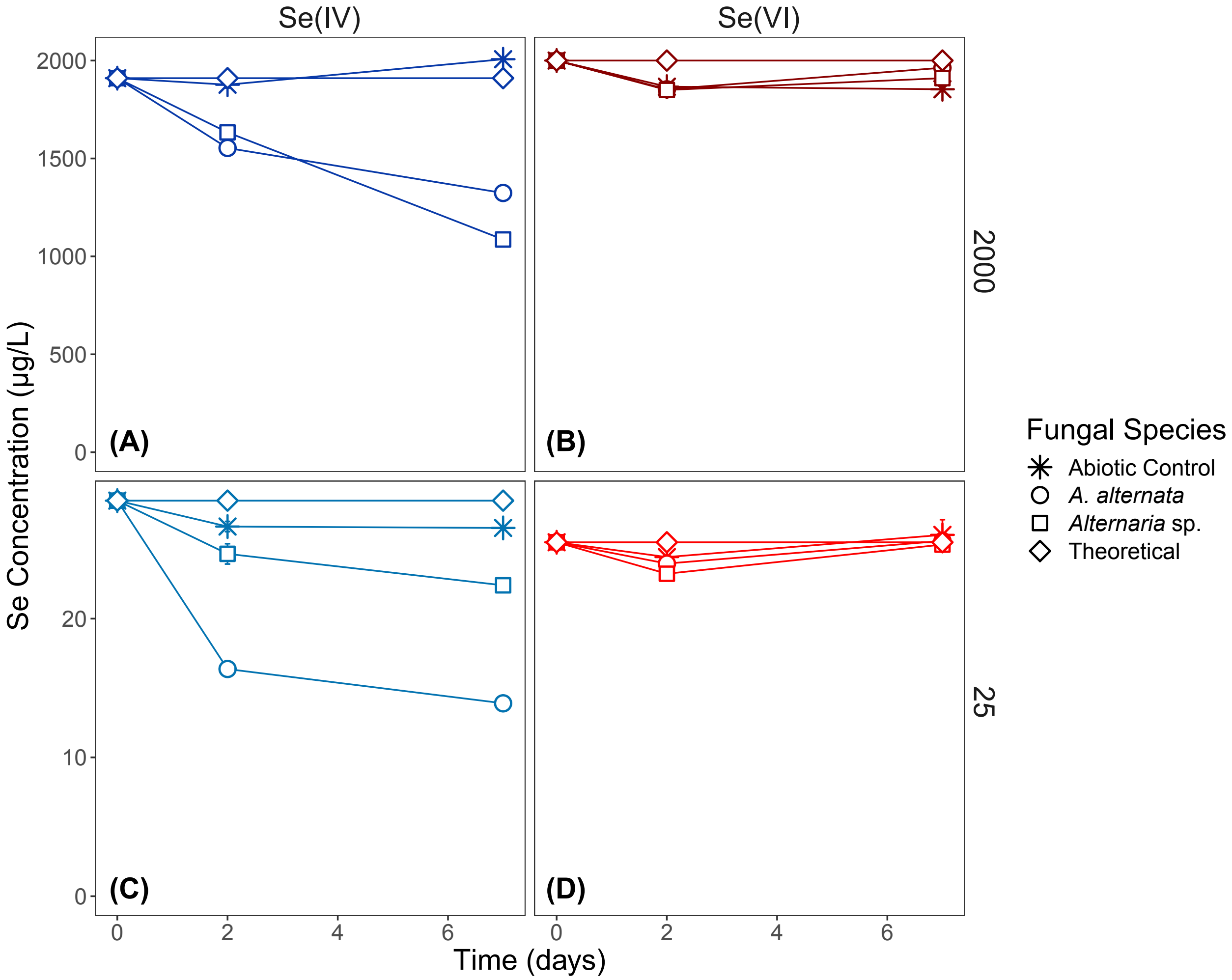

Supplementary Figure 3. Glycerin-amended media aqueous chemistry data for 2000 µg/L Se(IV and VI) (A, B) and 25 µg/L Se(IV and VI) (C, D). Error bars represent the standard deviation calculated from duplicates. For duplicates, data points represent calculated means and error bars represent actual values measured for each duplicate. Symbols without error bars have a standard deviation that is smaller than the symbol size. Theoretical calculations were made using the starting concentration of total Se measured, and assuming that under ideal abiotic conditions, the amount and volume of Se removed during sampling would be replaced when adding fresh media or wastewater back into the flask.

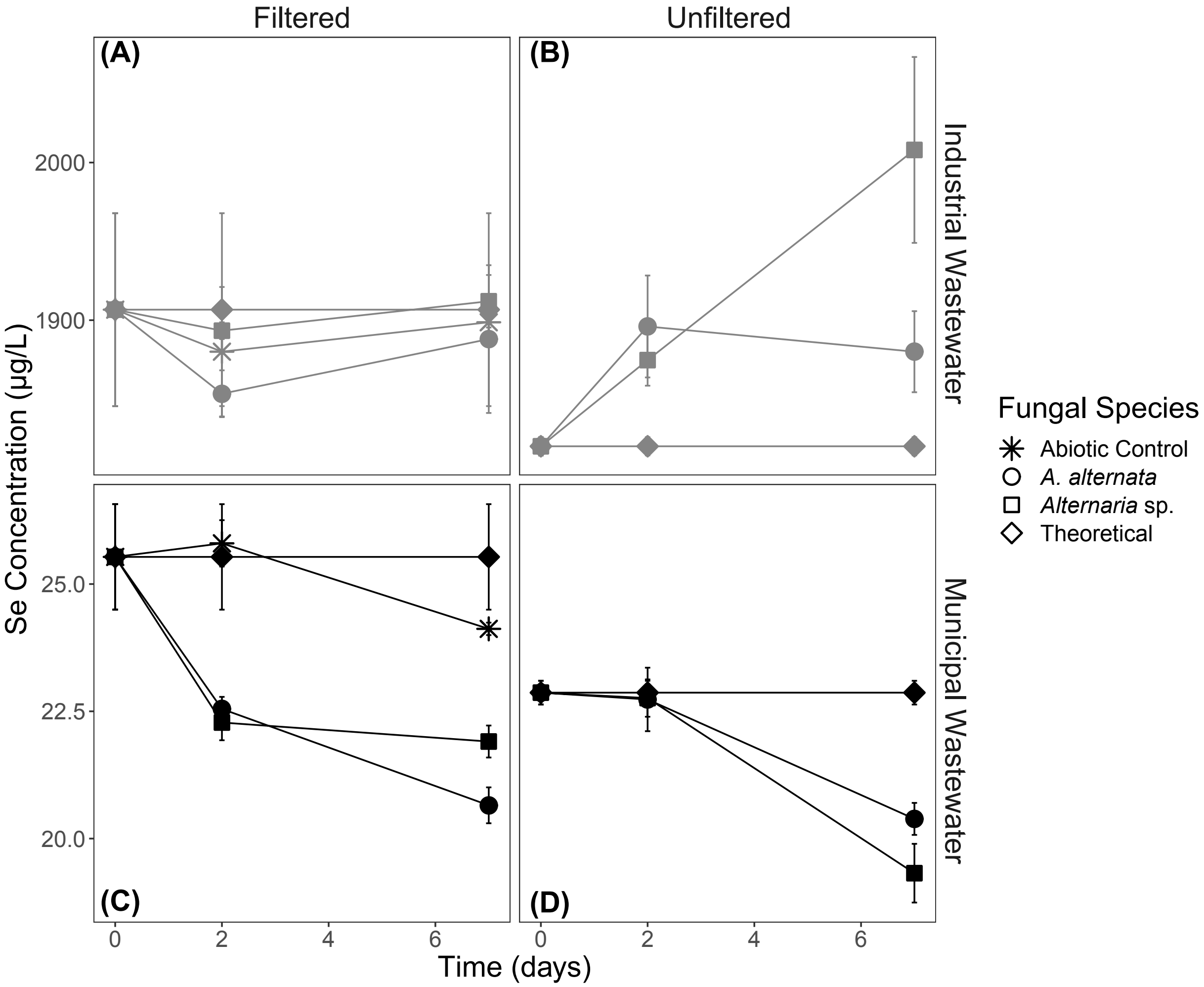

Supplementary Figure 4. Aqueous phase total Se concentrations over time in unamended WWI (A, B) and WWM (C, D) filtered and unfiltered experiments. Error bars represent the standard deviation calculated from triplicates. Symbols without error bars have a standard deviation that is smaller than the symbol size.

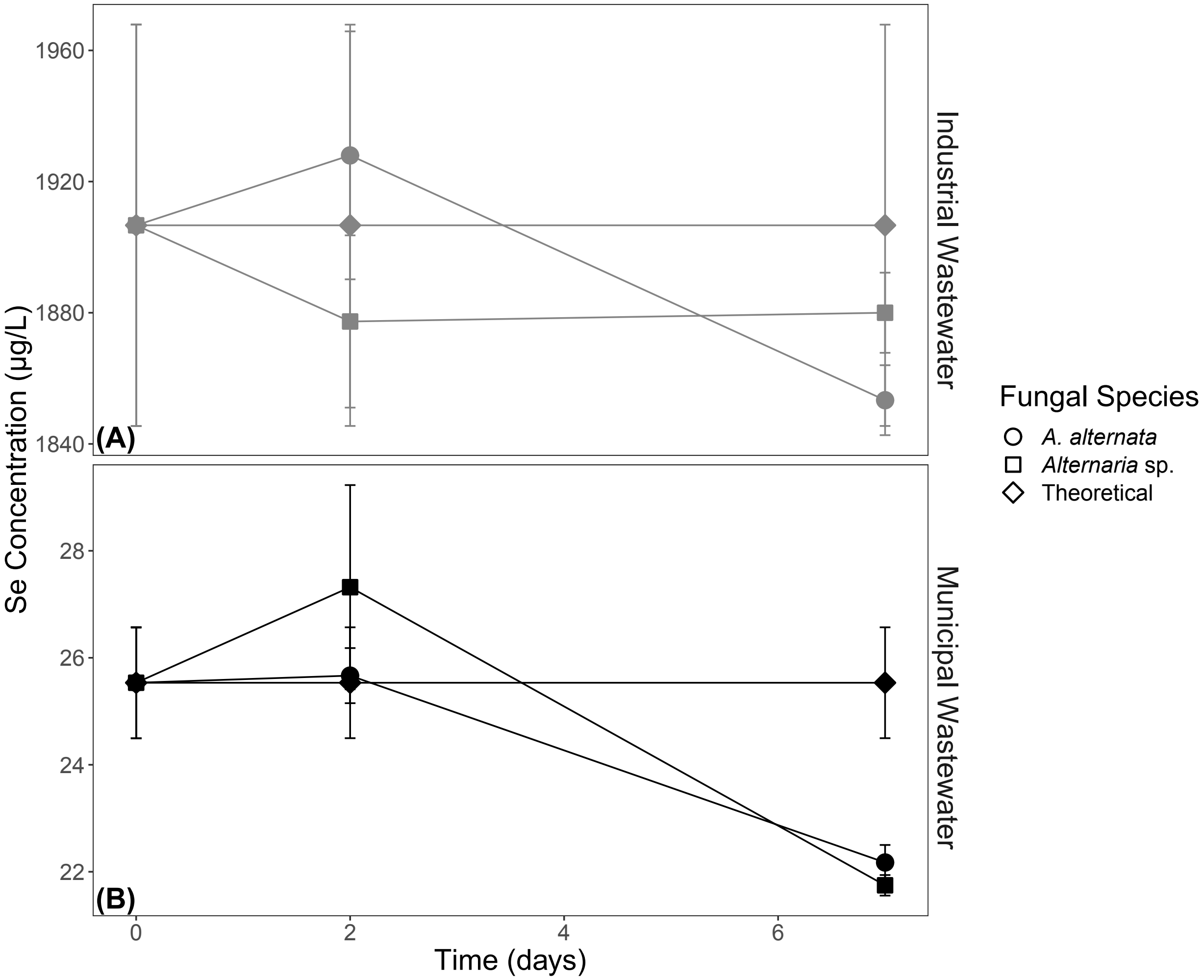

Supplementary Figure 5. Aqueous phase total Se concentrations over time in unamended WWI (A) and WWM (B) killed control experiments. Error bars represent the standard deviation calculated from triplicates. Symbols without error bars have a standard deviation that is smaller than the symbol size.

2.2 Supplementary Tables

Supplementary Table 1. Available Industrial and Municipal Wastewater Chemistry

Supplementary Table 2. Aqueous Se ICP-MS data

Supplementary Table 3. Solid-associated Se data

Supplementary Table 4. Biomass weight data

Supplementary Table 5. Summary of analysis of variance (ANOVA)
